# Three-tier regulation of cell number plasticity by neurotrophins and Tolls in *Drosophila*

**DOI:** 10.1083/jcb.201607098

**Published:** 2017-05-01

**Authors:** Istvan Foldi, Niki Anthoney, Neale Harrison, Monique Gangloff, Brett Verstak, Mohanakarthik Ponnadai Nallasivan, Samaher AlAhmed, Bangfu Zhu, Mark Phizacklea, Maria Losada-Perez, Marta Moreira, Nicholas J. Gay, Alicia Hidalgo

**Affiliations:** 1 NeuroDevelopment Group, School of Biosciences, University of Birmingham, Birmingham B15 2TT, England, UK; 2 Department of Biochemistry, University of Cambridge, Cambridge CB2 1GA, England, UK

## Abstract

Cell number plasticity is coupled to circuitry in the nervous system, adjusting cell mass to functional requirements. In mammals, this is achieved by neurotrophin (NT) ligands, which promote cell survival via their Trk and p75^NTR^ receptors and cell death via p75^NTR^ and Sortilin. *Drosophila* NTs (DNTs) bind Toll receptors instead to promote neuronal survival, but whether they can also regulate cell death is unknown. In this study, we show that DNTs and Tolls can switch from promoting cell survival to death in the central nervous system (CNS) via a three-tier mechanism. First, DNT cleavage patterns result in alternative signaling outcomes. Second, different Tolls can preferentially promote cell survival or death. Third, distinct adaptors downstream of Tolls can drive either apoptosis or cell survival. Toll-6 promotes cell survival via MyD88–NF-κB and cell death via Wek-Sarm-JNK. The distribution of adaptors changes in space and time and may segregate to distinct neural circuits. This novel mechanism for CNS cell plasticity may operate in wider contexts.

## Abstract (precis)

A three-tier mechanism involving distinct neurotrophin family ligand forms, different Toll receptors, and different adaptors regulates both cell survival and death. This rich mechanism confers cell number plasticity and could underlie structural plasticity in the nervous system and structural integrity, homeostasis, and regeneration in wider contexts.

## Introduction

Balancing cell death and cell survival enables structural plasticity and homeostasis, regeneration, and repair and fails in cancer and neurodegeneration. In the nervous system, cell number plasticity is linked to neural circuit formation, adjusting neuronal number to functional requirements (Levi-Montalcini, 1987). In mammals, the neurotrophin (NT) protein family—NGF, brain-derived neurotrophic factor (BDNF), NT3, and NT4—regulates neuronal number through two mechanisms. First, full-length pro-NTs, comprised of a disordered prodomain and a cystine-knot (CK) domain, induce cell death; in contrast, mature NTs formed of CK dimers promote cell survival (Lu et al., 2005). Second, pro-NTs bind p75^NTR^ and Sortilin receptors, inducing apoptosis via JNK signaling, whereas mature NTs bind p75^NTR^, promoting cell survival via NF-κB (Carter et al., 1996) and TrkA, B, and C, promoting cell survival via PI3K/AKT and MAPK/ERK (extracellular signal-related kinase; Lu et al., 2005). As the NTs also regulate connectivity and synaptic transmission, they couple the regulation of cell number to neural circuitry and function, enabling structural brain plasticity (Lu et al., 2005; Minichiello, 2009; Park and Poo, 2013). There is abundant evidence that cell number plasticity occurs in *Drosophila melanogaster* central nervous system (CNS) development, with neurotrophic factors including NTs and mesencephalic astrocyte-derived neurotrophic factor (MANF; Zhu et al., 2008; Palgi et al., 2009), but fruit flies lack p75^NTR^ and Trk receptors, raising the question of how this is achieved in the fly. Finding this out is important, as it could lead to novel mechanisms of structural plasticity for both flies and humans.

The *Drosophila* NTs (DNTs) Spätzle (Spz), DNT1, and DNT2 share with mammalian NTs the characteristic structure of a prodomain and a conserved CK of 13–15 kD, which forms a disulfide-linked dimer (Hoffmann et al., 2008a,b; Zhu et al., 2008; Arnot et al., 2010; Hepburn et al., 2014). Spz resembles NGF biochemically and structurally, and the binding of its Toll-1 receptor resembles that of NGF to p75^NTR^ (DeLotto and DeLotto, 1998; Mizuguchi et al., 1998; Arnot et al., 2010; Lewis et al., 2013; Hepburn et al., 2014). *DNT1* (also known as *spz2*) was discovered by homology to *BDNF*, and *DNT2* (also known as *spz5*) as a paralogue of *spz* and *DNT1* (Parker et al., 2001; Zhu et al., 2008). DNT1 and 2 promote neuronal survival, and DNT1 and 2, Spz, and Spz3 are required for connectivity and synaptogenesis (Zhu et al., 2008; Sutcliffe et al., 2013; Ballard et al., 2014). Spz, DNT1, and DNT2 are ligands for Toll-1, -7, and -6, respectively, which function as NT receptors and promote neuronal survival, circuit connectivity, and structural synaptic plasticity (Weber et al., 2003; Zhu et al., 2008; McIlroy et al., 2013; Sutcliffe et al., 2013; Ward et al., 2015; McLaughlin et al., 2016). Tolls belong to the Toll receptor superfamily, which underlies innate immunity (Imler and Zheng, 2004; Leulier and Lemaitre, 2008). There are nine *Toll* paralogues in flies, of which only Toll-1, -5, -7, and -9 are involved in immunity (Tauszig et al., 2000; Leulier and Lemaitre, 2008). Tolls are also involved in morphogenesis, cell competition, and epidermal repair (Halfon et al., 1995; Yagi et al., 2010; McIlroy et al., 2013; Ballard et al., 2014; Carvalho et al., 2014; Meyer et al., 2014; Paré et al., 2014; Ward et al., 2015). Whether DNTs and Tolls can balance cell number plasticity is unknown.

Like the p75^NTR^ receptor, Toll-1 activates NF-κB (a potent neuronal prosurvival factor with evolutionarily conserved functions also in structural and synaptic plasticity) signaling downstream (Hoffmann and Reichhart, 2002; Mattson and Meffert, 2006; Gutierrez and Davies, 2011). Toll-1 signaling involves the downstream adaptor MyD88, which forms a complex with Tube and Pelle (Horng and Medzhitov, 2001; Tauszig-Delamasure et al., 2002; Gay and Gangloff, 2007). Activation of Toll-1 triggers the degradation of the NF-κB inhibitor Cactus, enabling the nuclear translocation of the NF-κB homologues Dorsal and Dorsal-related immunity factor (Dif), which function as transcription factors. Other Tolls have also been suggested to activate NF-κB (McIlroy et al., 2013; Meyer et al., 2014). However, only Toll-1 has been shown to bind MyD88 (Tauszig-Delamasure et al., 2002), raising the question of how the other Tolls signal in flies.

Whether Tolls regulate cell death is also obscure. Toll-1 activates JNK, causing apoptosis, but its expression can also be activated by JNK to induce nonapoptotic cell death (Liu et al., 2015; Wu et al., 2015a,b). Toll-2, -3, -8, and -9 can induce apoptosis via NF-κB and dSarm independently of MyD88 and JNK (Meyer et al., 2014). However, in the CNS, dSarm induces axonal degeneration, but there is no evidence that it can promote apoptosis in flies (Osterloh et al., 2012). In other animals, Sarm orthologues are inhibitors of Toll signaling and MyD88 (Carty et al., 2006; Yuan et al., 2010), but there is no evidence that dSarm is an inhibitor of MyD88 in *Drosophila*. Thus, whether or how Tolls may regulate apoptosis in flies is unclear.

In the mammalian brain, Toll-like receptors (TLRs) are expressed in neurons, where they regulate neurogenesis, apoptosis, and neurite growth and collapse in the absence of any insult (Okun et al., 2011). However, their neuronal functions have been little explored, and their endogenous ligands in neurons remain unknown.

Because Toll-1 and p75^NTR^ share common downstream signaling pathways and p75^NTR^ can activate NF-κB to promote cell survival and JNK to promote cell death, we asked in this study whether the DNTs and their Toll receptors could have dual roles controlling cell survival and death in the *Drosophila* CNS.

## Results

### Different processing for each DNT ligand

**Figure 1.**
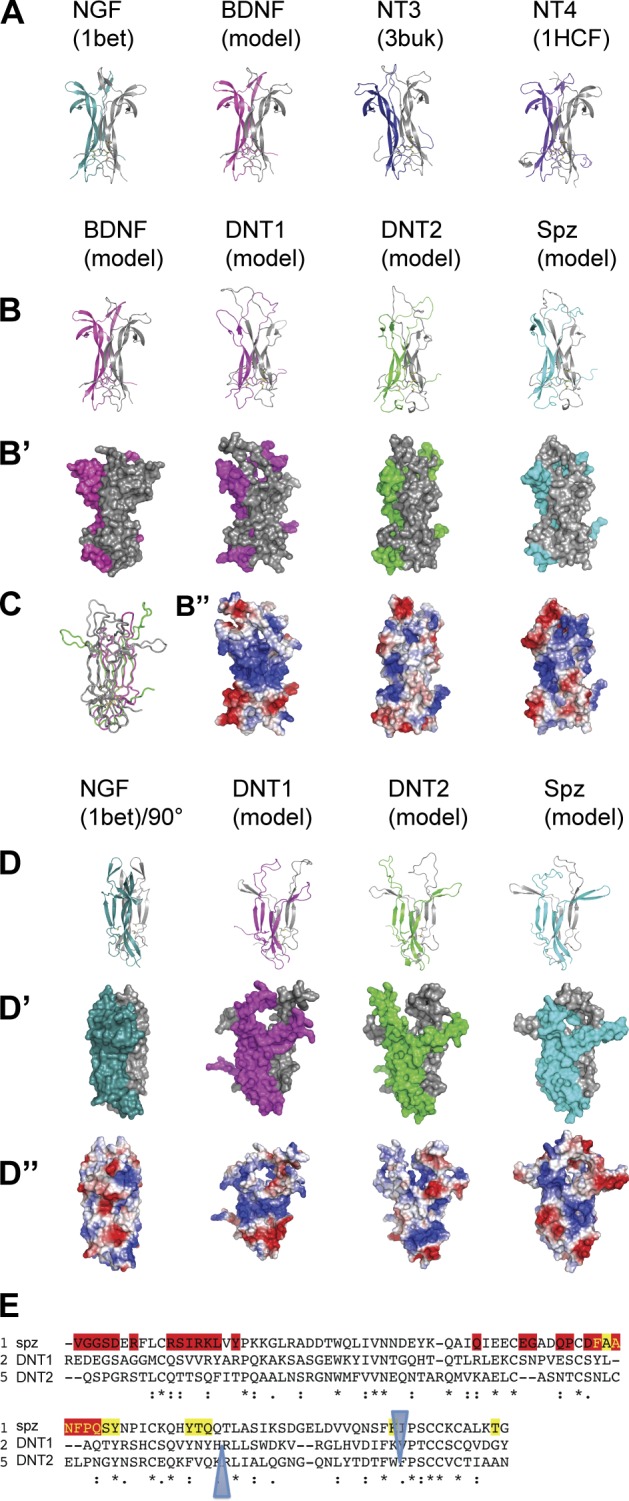
**Structural models of DNTs compared with Spz and mammalian NTs.** (A–D) Crystal structures and homology models of mammalian NTs and DNTs compared in the same orientation relative to the CK. PDB code 1BET corresponds to the crystal structure of NGF as determined by McDonald et al. (1991), and 3BUK and 1HCF correspond to the crystal structures of NT3 in complex with p75 (Gong et al., 2008) and NT4/5 bound to TrkB (Banfield et al., 2001). Representations in A, B, and D are cartoons, in B’ and D’ are molecular surfaces color coded by protomers, and in B’’ and D’’ are surface charge distributions with gradients from red to white to blue, corresponding with electronegative to neutral to positively charged molecular surfaces. Mammalian NTs (A and B, BDNF; D, NGF) and DNTs (B and D) have conserved CK but deviating β-hairpin wings. Compare Spz and NGF (A and D) and DNT2 (green and gray) with BDNF (magenta and gray) in ribbon representation (C). (E) Spz residues mediating Toll-1 binding are not conserved among DNTs. Clustal annotations marked by asterisks were used for identical residues, and colons mark increased similarity between residues. Spz residues from the proximal Spz chain interfacing with Toll-1 are in red, Spz residues from the distal Spz chain are in yellow, and blue triangles indicate conserved areas. Of 33 Toll-contact residues in Spz, only 11 are conserved in DNT1 and 2, with a single identical residue Tyr^64^ in Spz.

Using 3D structural modeling based on the crystal structure of Spz (Lewis et al., 2013), we compared the mature CK domains of DNTs with those of mammalian NTs. They all share the structurally conserved CK unique to the NT family and distinct from those of other growth factors, with the characteristic arrangement of antiparallel β sheets and disulfide bridges (Fig. 1, A–D). The overhanging wings are out of phase by 90° in *Drosophila* versus mammalian ligands, possibly reflecting interactions with different receptor types (Fig. 1, B–D). The receptor-binding interface of Spz is not evolutionarily conserved in DNT1 or 2, suggesting distinct receptor affinities (Fig. 1 E). Thus, Spz, DNT1, and DNT2 are NT ligands with distinctive features.

**Figure 2.**
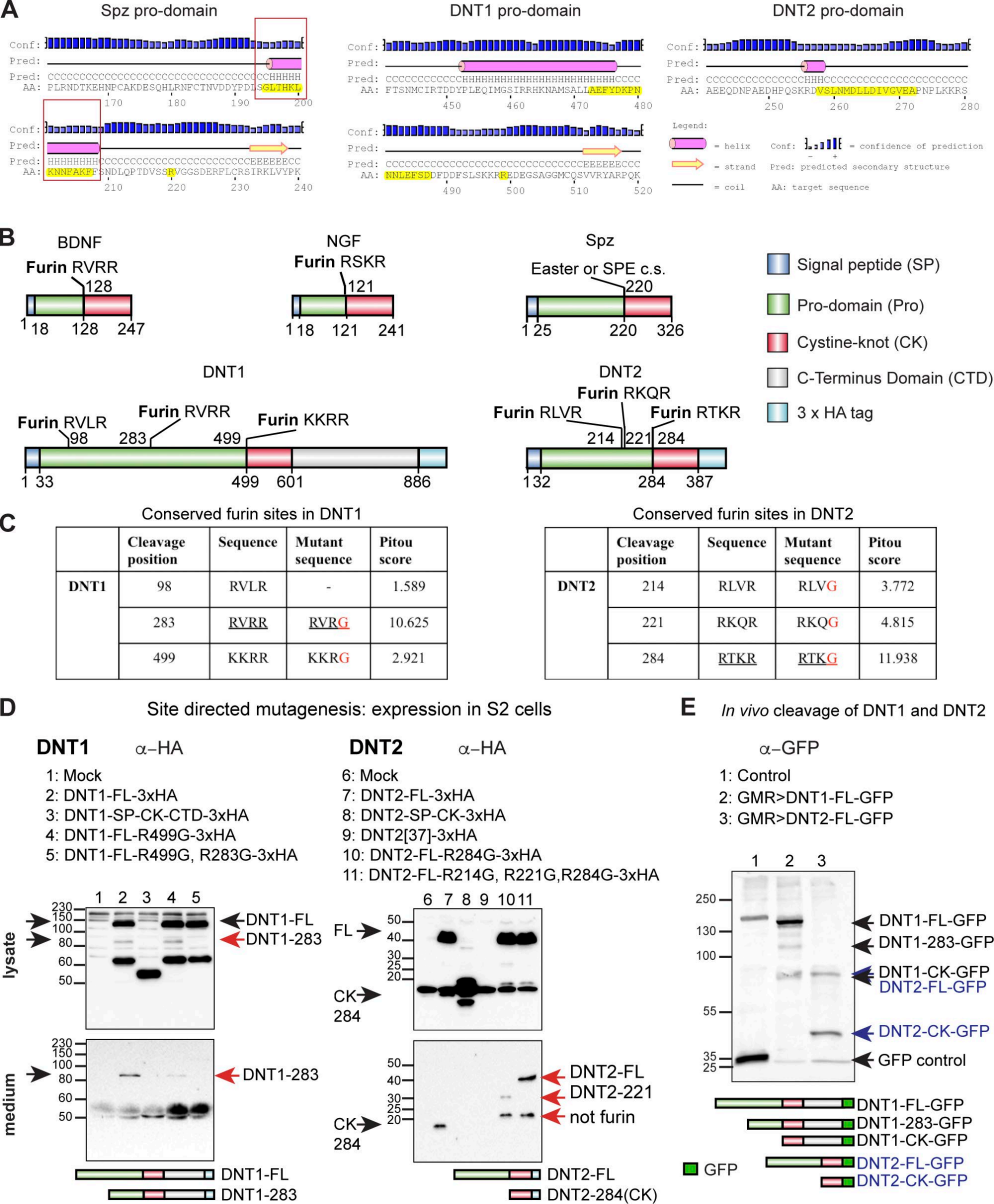
**DNT1 and 2 are cleaved by conserved furin proteases.** (A) The prodomain α-helix of Spz (boxes) required to activate Toll-1 is not conserved in DNT1 and 2. Yellow highlights indicate corresponding sequences that are not conserved. (B) The prodomains of DNT1 and 2 but not Spz have conserved furin sites. c.s., cleavage site. (C) Site-directed mutagenesis of furin sequences. Red letters mark amino acid substitutions. (D) Mutant DNT1-FL-HA and DNT2-FL-HA forms expressed in S2 cells and visualized in Western blots with anti-HA from lysate and secreted medium. Black arrows indicate normal forms, and red arrows indicate mutant products. (E) Anti-GFP Western blot upon overexpression of C-terminally tagged DNTs in the retina with *GMR-GAL4* shows that furin cleavage occurs in vivo (black arrows: DNT1, blue arrows: DNT2). Molecular masses on the left of each blot are given in kilodaltons.

The prodomains have distinctive features too. The prodomains of Spz and DNT2 are disordered coils, whereas that of DNT1 has helices, suggesting a globular structure (Figs. 2 A and S1). The DNT1 prodomain is also twice as long as that of DNT2. The prodomain of Spz has an α-helix just upstream of the Easter cleavage site, which undergoes a conformational change upon cleavage, essential for the activation of Toll (Arnot et al., 2010). This sequence is not conserved in the prodomains of the mammalian NTs nor in DNT1 and 2 (Fig. 2 A). This suggests that the activation mechanism of Toll by Spz is unique and distinct from those of Toll-6 and -7 by DNT2 and 1, respectively.

Mammalian pro-NTs are cleaved intracellularly by furin proteases or extracellularly by serine proteases (e.g., BDNF; Fig. 2 B). Spz is only secreted full length and is cleaved extracellularly by the serine proteases Easter or Spz processing enzyme (Hoffmann and Reichhart, 2002). Furin sites were absent from the Spz prodomain, but several highly conserved sites were found in DNT1 and 2 (Fig. 2 B). In vivo overexpression of mature *Spz-CK*, *DNT1-CK*, and *DNT2-CK* is functional and rescues the respective mutant phenotypes (Ligoxygakis et al., 2002; Hu et al., 2004; Zhu et al., 2008; Sutcliffe et al., 2013). However, S2 cells transfected with DNT1–CK–C-terminal domain (CTD) tagged with 3×HA (*DNT1-CK-CTD-HA*) and *DNT2-CK-HA* did not secrete mature DNTs to the S2 cell medium (Fig. 2 D, lanes 3 and 8). This either suggests that the prodomain is required for trafficking in S2 cells or that S2 cells do not behave like neurons do in vivo. S2 cells transfected with wild-type full-length (FL) *DNT1-FL-HA* did not secrete DNT1-FL either but instead secreted a product truncated at the R283 site (Fig. 2 D, lane 2), suggesting that cleavage occurs naturally at this site. In contrast, S2 cells expressing *DNT2-FL-HA* invariably secreted the mature CK form of 15 kD (Fig. 2 D, lane 7). To test whether the conserved furin sites were responsible for these cleavage profiles, we performed site-directed mutagenesis of the furin sequences in HA-tagged DNT1 and 2 (Fig. 2 C). DNT1 lacking the furin site at R499 still secreted a product cleaved at R283, but no secreted protein was detected when both R499 and R283 were mutagenized (Fig. 2 D, lanes 4 and 5). Thus, the DNT1 furin site at R283, which is the most conserved, is functional. Mutagenesis of the DNT2 furin site R284 resulted in the secretion of two products of 30 kD and 18 kD (Fig. 2 D, lane 10). The 30-kD product corresponds to cleavage at site R214 or R221, implying that cleavage at these sites is unlikely to occur naturally or that cleavage at R284 predominates. The 18-kD product was not detectable in the media expressing wild-type DNT2, suggesting that it does not occur naturally and is the result of nonfurin cleavage. Mutagenizing R214, R221, and R284 sites resulted in the secretion of DNT2-FL-HA from S2 cells, showing that DNT2 can be secreted full length (Fig. 2 D, lane 11). These findings showed that the DNT2 furin cleavage site at R284 is functional and is the predominant cleavage site.

To test whether similar DNT processing occurs in vivo*,* we overexpressed in the retina (with *GMR-GAL4*) full-length forms tagged at the C termini with GFP and visualized the resulting products with anti-GFP in Western blots. DNT1 was predominantly found in full-length form and also cleaved at furin sites at 98 (less abundant), 283 (pro-DNT1), and 499 (DNT1-CK-CTD; Fig. 2 E). DNT2 was found full length, but predominantly in mature form (DNT2-CK; Fig. 2 E). These data show that in vivo, DNTs are cleaved by furins and can be found in both pro- and mature forms.

To conclude, each DNT has unique features. DNT1 is more likely found in pro- form than DNT2, and DNT2 is more likely found in mature form. Ultimately, the forms secreted in vivo will depend on the expression profile of proteases and will be context dependent. The distinct processing mechanisms of Spz, DNT1, and DNT2 suggest functional differences.

### Pro-DNT1 activates proapoptotic and mature DNT prosurvival pathways

To ask whether different DNT forms could have distinct functions, we tested whether they could activate proapoptotic or prosurvival signaling pathways.

**Figure 3.**
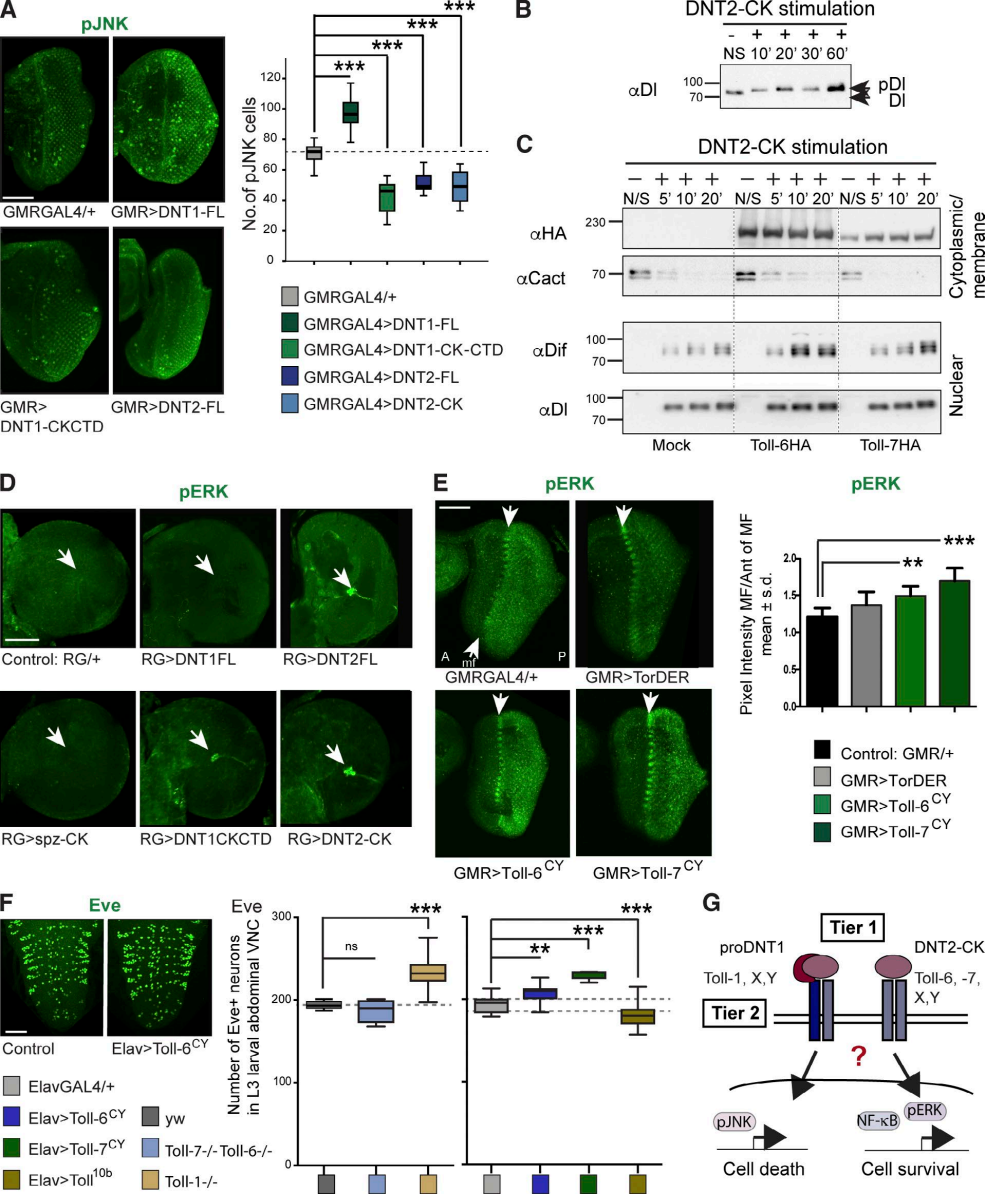
**DNTs and Tolls activate proapoptotic and prosurvival pathways.** (A) Overexpression of DNTs in larval retina with *GMR-GAL4*–altered pJNK activation. The box plot graph depicts a one-way analysis of variance: ***, P < 0.001; Dunnett’s post-hoc test. (B) Stimulation of S2 cells with purified DNT2-CK–induced Dorsal phosphorylation. (C) Stimulation of S2 cells with DNT2-CK provoked the degradation of the cytoplasmic inhibitor Cactus (αCact) and the nuclear translocation of Dif (αDif) and Dorsal (αDI), particularly in *Toll-6*– or *-7*–transfected cells. Molecular masses are given in kilodaltons. Dotted lines indicate that intervening lanes have been spliced out. (D) Overexpression of *DNT1-CK-CTD* and *DNT2-CK*, but not *DNT1-FL* or *spz-CK*, activated ERK (arrows) in *RG-GAL4* neurons of the larval optic lobe. *n* = 5–11. (E) Overexpression of activated T*oll-6^CY^* and -*7^CY^* in the retina increased pERK (arrows indicate morphogenetic furrow [mf]). *GMR-GAL4>TORDER* is a positive control. Error bars display SD (s.d.). One-way analysis of variance: P < 0.0001; Dunnett’s post-hoc test. *n* = 8–13. A, anterior; P, posterior. (F) Distinct effect of loss and gain of function for Tolls in Eve^+^ neuron numbers in larvae. Dashed lines indicate the median (left graph) or 50% of the data distribution in controls (right graph). One-way analysis of variance: ***, P < 0.0001; Dunnett’s post-hoc test. *n* = 5–22. ns, not significant. Asterisks on graphs indicate post-hoc multiple comparisons corrections: **, P < 0.01; ***, P < 0.001. > indicates GAL4/UAS. Bars, 50 µm. (G) Different ligand forms and Toll receptors can induce either cell survival or death. For genotypes, statistical details, and sample sizes, see Table S2.

Overexpression of mature DNT1 and 2 promotes cell survival in embryos (Zhu et al., 2008). In mammals, apoptosis is activated by pro-NTs binding p75^NTR^ and activating JNK (Roux and Barker, 2002). Thus, we asked whether the different DNT forms activate JNK signaling, visualized using antiphospho-JNK antibodies. Overexpression of *DNT1-CK-CTD*, *DNT2-CK*, or *DNT2-FL* in the retina reduced the number of pJNK^+^ cells compared with controls, whereas overexpression of *DNT1-FL* increased pJNK^+^ cell number (Fig. 3 A). Most likely, DNT2-FL was cleaved intracellularly and secreted as mature CK instead (Fig. 2, D and E). Thus, pro-DNT1 can activate the JNK proapoptotic signaling pathway.

We next tested whether DNTs can activate the prosurvival pathways NF-κB and ERK. Stimulating S2 cells with purified mature DNT2-CK induced the phosphorylation of Dorsal (i.e., activation; Fig. 3 B). We also transfected S2 cells with *Toll-6* or *-7*, stimulated them with purified mature DNT2-CK, and tested whether it triggered the nuclear translocation of Dorsal or Dif, thus activating NF-κB signaling. Subcellular fractionation revealed that DNT2 induced the degradation of the NF-κB inhibitor Cactus in the cytoplasm and the nuclear translocation of both Dorsal and Dif (Figs. 3 C and S2 A). These data demonstrate that mature DNT2-CK activates NF-κB signaling. Stimulation with DNT2-CK also activated signaling in nontransfected control cells (Fig. 3 C). Because S2 cells express multiple Tolls, but not Toll-6 (Fig. S2 B), this means that DNT2 can also bind other Toll family receptors. In fact, DNT1 binds Toll-7 and DNT2 binds Toll-6 (McIlroy et al., 2013), but DNT1 could also bind Toll-6 and DNT2 could also bind Toll-7 (Fig. S3). Thus, binding of DNT1 and 2 to Toll-6 and -7 is promiscuous. Importantly, both Cactus degradation and nuclear translocation of Dorsal and Dif induced by DNT2 were more pronounced in transfected cells than in mock controls (Fig. 3 C). This shows that Toll-6 and -7 activate NF-κB signaling downstream of DNT2.

To test whether DNTs, Toll-6, and Toll-7 could activate ERK, we overexpressed them and visualized activated antiphospho-ERK. Overexpression of either *DNT1-FL* or mature *spz-CK* in neurons of the larval brain optic lobe (with *RG-GAL4*) did not activate ERK signaling (Fig. 3 D). In contrast, overexpression of *DNT1-CK-CTD* and *DNT2-CK* did (Fig. 3 D). DNT2-FL also activated ERK, but as shown in Fig. 2 (D and E), DNT2 is readily cleaved before secretion. Thus, mature DNT1 and 2 (but not Spz) can activate ERK. Furthermore, overexpression of activated forms of *Toll-6^CY^* and *Toll-7^CY^* (McIlroy et al., 2013) in retinae significantly increased pERK levels (Fig. 3 E). Thus, Toll-6 and -7 activate ERK. Collectively, these data show that DNT1 and 2 can activate the prosurvival signaling pathways NF-κB and ERK via Toll-6 and -7 and that pro-DNT1 can activate the proapoptotic JNK pathway.

To test whether distinct Toll receptors might differentially regulate neuronal number, we asked whether Eve^+^ neurons were affected by loss or gain of function for Tolls in third instar larvae ventral nerve cords (VNCs). *Toll-7^P8^/ Toll-7^P114^; Toll-6^26^/Toll-6^31^* double mutant larvae had slightly fewer Eve^+^ neurons than wild type, but *Toll-1^r3^/Toll-1^r444^* mutants had more (Fig. 3 F). Conversely, overexpression of constitutively active *Toll-6^CY^* and *Toll-7^CY^* in neurons (with *Elav-GAL4*) increased Eve^+^ neuron number (Fig. 3 F), whereas constitutively active *Toll-1^10b^* decreased it (Fig. 3 F). Thus, Toll-6 and -7 promote cell survival as previously described (McIlroy et al., 2013), but Toll-1 can be proapoptotic. Distinct Toll receptors and the potential formation of heterodimers between different Toll receptors might switch the response to DNTs from cell survival to cell death (Fig. 3 G).

### Toll-6 activates prosurvival signaling in the CNS via MyD88

**Figure 4.**
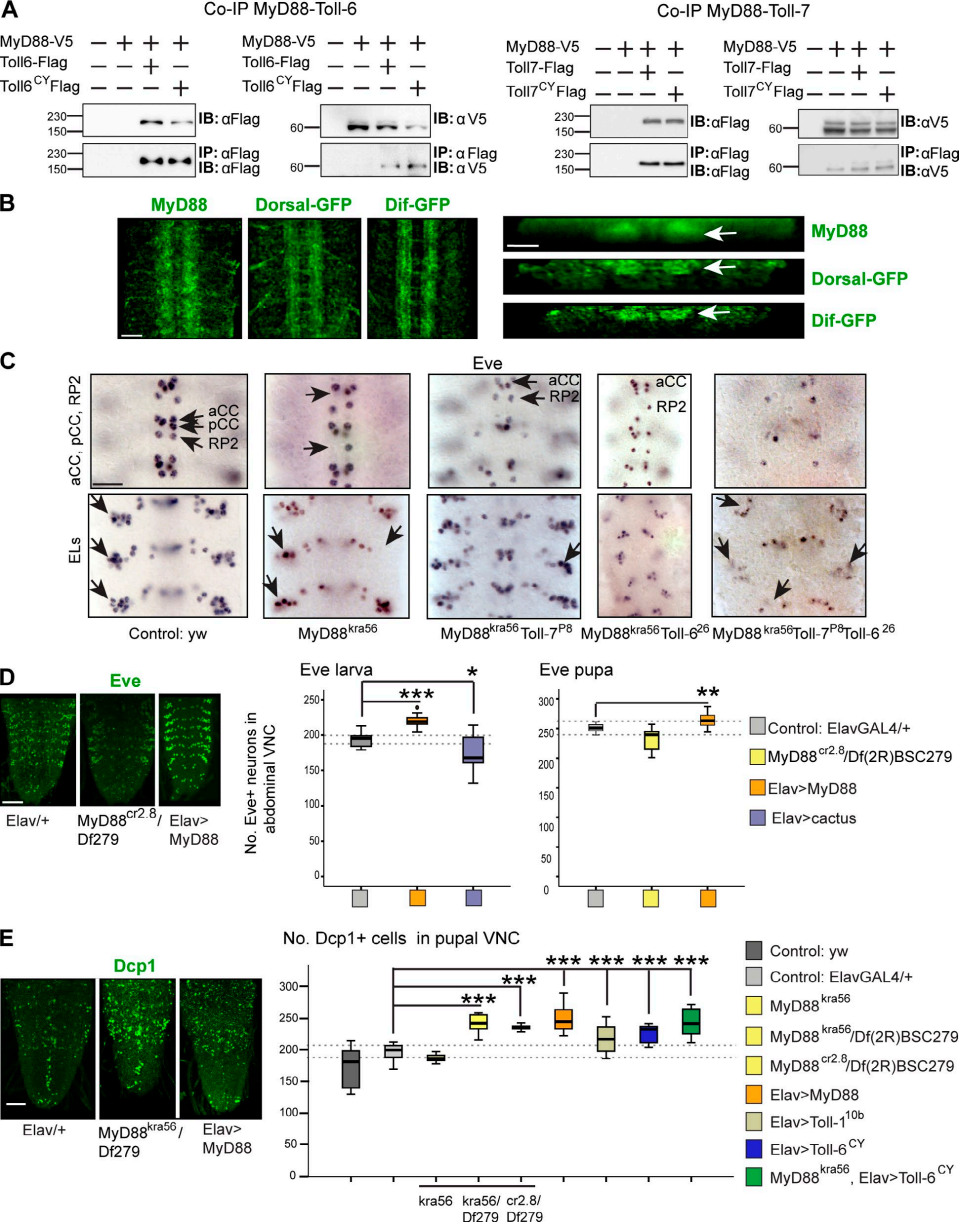
**Toll-6 promotes cell survival via MyD88.** (A) Coimmunoprecipitations showing that MyD88-V5 bound Toll-6–Flag and Toll-7–Flag and activated Toll-6^CY^–Flag and Toll-7^CY^–Flag. IB, immunoblot; IP, immunoprecipitation. Molecular masses are given in kilodaltons. (B) Anti-MyD88 and exon trap reporters Dorsal-GFP and Dif-GFP visualized with anti-GFP are distributed throughout the embryonic CNS neuropile. Left, horizontal views; right, transverse sections; white arrows indicate reporter distribution within the neuropile. (C) Loss of Eve^+^ neurons (arrows) in the CNS in *MyD88^kra56^ Toll-7^P8^ Toll-6^26^* triple mutant embryos. For quantification, see Fig. S4. aCC, anterior corner cell; EL, Eve lateral. (D) Altering MyD88 signaling affects Eve^+^ neuron number. Dashed lines indicate 50% (left graph) or 100% (right graph) data distirbution in controls. Box plots: larvae, one-way analysis of variance, P < 0.001, Dunnett’s post-hoc test; pupae, Welch’s analysis of variance, P < 0.01, Dunnett’s post-hoc test. *n* = 8–12. (E) Apoptotic cells visualized with anti-Dcp1 in white pupal VNCs and counted automatically with DeadEasy software. Box plot: Welch’s analysis of variance, P < 0.001, Dunnett’s post-hoc test. *n* = 5–16. Asterisks on graphs indicate post-hoc multiple comparisons corrections: *, P < 0.05; **, P < 0.01; ***, P < 0.001. For statistical details, see Table S2. > indicates GAL4/UAS. Bars: (B and C) 100 µm; (D and E) 50 µm.

The finding that Toll-6 and -7 could initiate signaling suggested the involvement of the MyD88 adaptor. However, no interactions between Tolls other than Toll-1 and MyD88 had been previously detected (Tauszig-Delamasure et al., 2002). To test whether Toll-6 or -7 and MyD88 could form a signaling complex, S2 cells were cotransfected with native *Toll-6* or *-7* or activated *Toll-6^CY^* or *-7^CY^* tagged with Flag and *MyD88* tagged with *V5*. In coimmunoprecipitations, MyD88 copurified with Toll-6 and -7 as well as Toll-6^CY^ and Toll-7^CY^ (Fig. 4 A). Thus, both Toll-6 and -7 can bind MyD88. This physical interaction could occur in vivo, as, like Toll-6 and -7 (McIlroy et al., 2013), MyD88 protein was found throughout the embryonic CNS neuropile, and its endogenously tagged downstream targets Dorsal-GFP and Dif-GFP were as well (Fig. 4 B).

We next asked whether MyD88 is required for the CNS functions of Toll-6 and -7 by testing the effect of mutants on Eve, a reporter for Toll-6 neurons. All Eve^+^ neurons except posterior corner cell (pCC) and RP2 express *Toll-6* (McIlroy et al., 2013). *MyD88^kra56^* is a hypomorphic allele (Charatsi et al., 2003), ideal to test for phenotypic enhancement or suppression in genetic interactions. *MyD88^kra56^* mutants had a virtually normal embryonic CNS, but *MyD88^kra56^ Toll-6^26^* double mutants and *MyD88^kra56^ Toll-7^P8^ Toll-6^26^* triple mutants had fewer Eve^+^ neurons (Figs. 4 C and S4). This is consistent with MyD88 functioning downstream of Toll-6 and -7 in the CNS to maintain neuronal survival. In fact, overexpression of *MyD88* in all neurons (with *elav-GAL4*) increased Eve^+^ neurons both in third instar larvae and pupae (Fig. 4 D). Conversely, overexpression of *cactus* decreased Eve^+^ neuron numbers in larvae (Fig. 4 D), and loss of *MyD88* function also decreased Eve^+^ numbers in pupae (Fig. 4 D). Collectively, these data show that MyD88 is required for and can promote neuronal survival.

To verify this, we quantified the effects of altering *MyD88* function in apoptosis. *MyD88^kra56^* homozygotes are semilethal, with a lethality phase at pupariation, indicating this is a critical time for MyD88 function. Using anti–Death Caspase 1 (Dcp1), we counted all dying cells throughout the VNC of white pupae using adapted DeadEasy Caspase software (Forero et al., 2009). In *MyD88^kra56^* homozygotes, apoptosis levels did not differ from controls, but they increased in *MyD88^kra56^/DfBSC279* trans-heterozygotes (Fig. 4 E). We generated a *MyD88*-null allele using clustered regularly interspaced short palindromic repeats (CRISPR)/Cas9, *MyD88^cr2.8^*. Trans-heterozygous *MyD88^cr2.8^/Df(2R)BSC279* pupae also had increased apoptosis (Fig. 4 E). Thus, MyD88 is required for neuronal survival. Collectively, these data show that Toll-6 and -7 signal via the canonical MyD88 pathway to promote neuronal survival in the CNS.

However, overexpression of *MyD88* in all neurons also increased apoptosis in pupae (Fig. 4 E). This could occur downstream of Tolls, as overexpression of activated *Toll-6^CY^* or *Toll-1^10b^* also increased apoptosis in pupae (Fig. 4 E). Remarkably, the proapoptotic effect of Toll-6 was enhanced when overexpressed in a *MyD88^kra56^* mutant background (Fig. 4 E), suggesting that Toll-6 might induce apoptosis in pupae independently of MyD88.

These data raised two questions: How does MyD88 induce apoptosis? And how can Toll-6 induce apoptosis independently of MyD88?

### Toll-6 can induce apoptosis via the MyD88 inhibitor dSarm

**Figure 5.**
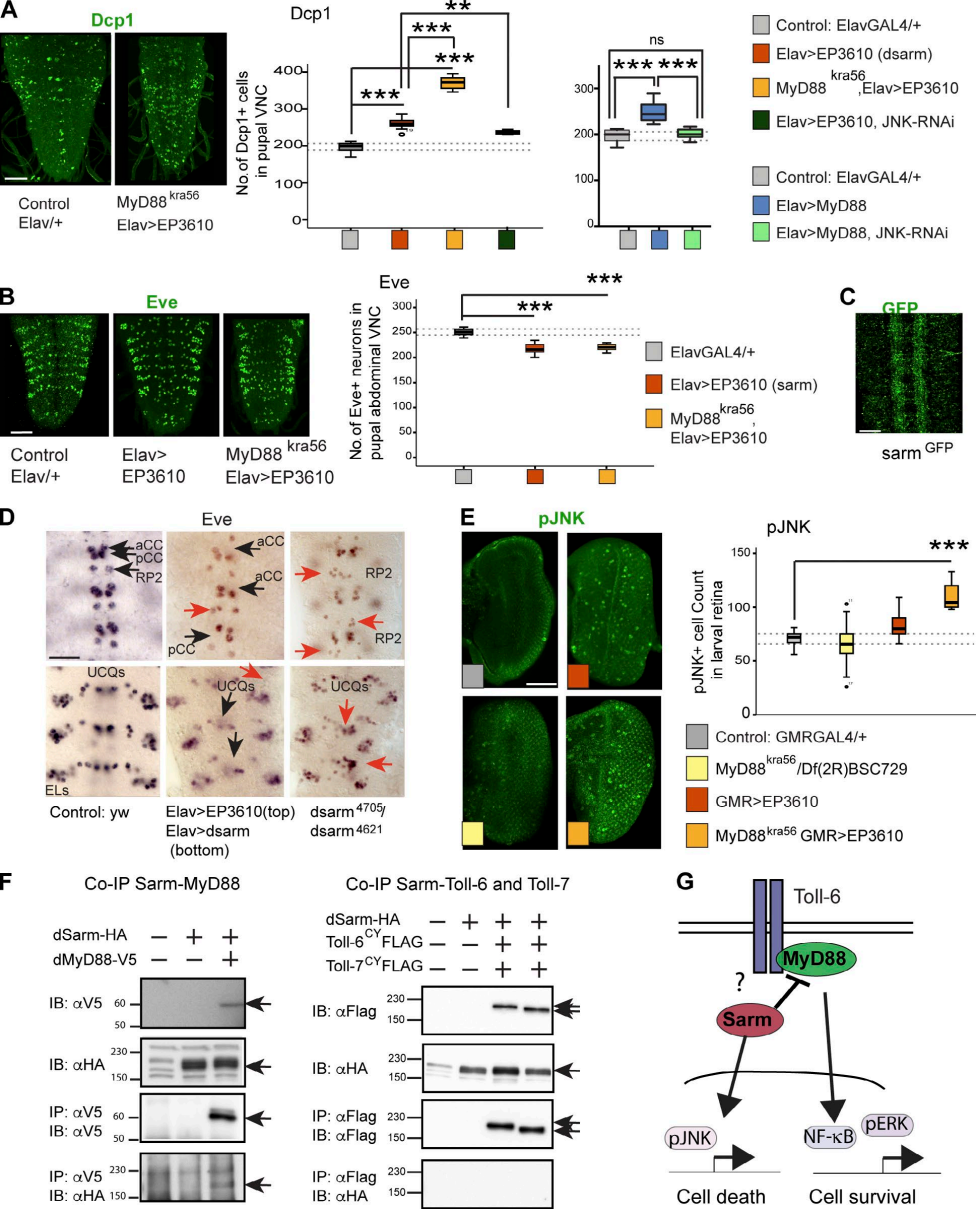
**dSarm antagonizes MyD88 and promotes apoptosis downstream of Toll-6.** (A) Apoptotic cells visualized with anti-Dcp1 in pupal VNCs and quantified with DeadEasy. Box plots: left, Welch’s analysis of variance, P < 0.0001, Bonferroni’s post-hoc test; right, one-way analysis of variance, P < 0.0001, Tukey’s post-hoc test. *n* = 9–16. ns, not significant. (B) Eve^+^ neuron numbers in the abdominal VNCs of L3 larvae are regulated by dSarm. Box plot: one-way analysis of variance, P < 0.0001, Dunnett’s post-hoc test. *n* = 9–12. (C) Anti-GFP in *dsarm^MI08854^-GFP* is distributed throughout the embryonic CNS neuropile. (D) Loss and gain of *dsarm* function affects Eve^+^ neuron numbers in embryos (black arrows indicate neuronal loss, and red arrows indicate supernumerary neurons). For quantification, see Fig. S4. aCC, anterior corner cell; EL, Eve lateral. (E) dSarm can activate JNK signaling, seen with anti-pJNK in the larval retina. Box plot: one-way analysis of variance, P < 0.001, Dunnett’s post-hoc test. *n* = 4–18. (F) Coimmunoprecipitation from S2 cells showing that dSarm binds MyD88, but does not bind Toll-6 or -7. Arrows point to relevant bands. IB, immunoblot; IP, immunoprecipitation. Molecular masses are given in kilodaltons. (G) dSarm inhibits MyD88 and activates JNK, promoting apoptosis. Asterisks on graphs indicate post-hoc multiple comparisons corrections: **, P < 0.01; ***, P < 0.001. See Table S2. > indicates GAL4/UAS. Bars: (A, C, and E) 50 µm; (B) 100 µm.

In mammals, Sarm1 inhibits MyD88 and can induce neuronal apoptosis (O’Neill and Bowie, 2007; Carlsson et al., 2016). Thus, we wondered whether *Drosophila dsarm* might be involved in proapoptotic signaling by Toll-6. We overexpressed *dsarm* in all neurons using *EP3610* flies, which drive expression of multiple *Ect4* isoforms (*Ect4* is a synonym of *dsarm*). *Elav>EP3610* increased apoptosis in pupal VNCs (Fig. 5 A). Remarkably, overexpression of *dsarm* in a *MyD88^kra56^* mutant background increased apoptosis further (Fig. 5 A). This showed that *dSarm* promotes apoptosis and antagonizes MyD88 function. Apoptosis led to neuronal loss, as overexpression of *dsarm* in normal or *MyD88* mutant pupae decreased Eve^+^ neuron number (Fig. 5 B). Because *sarm* mutants are embryonically lethal, to further verify this, we looked at the embryonic CNS. *dsarm* is expressed throughout the embryonic CNS, as visualized with a *dsarm^MIMIC^-GFP* reporter (Fig. 5 C). Overexpressing *dsarm* using either *EP3610* or a single *dsarm* isoform (Osterloh et al., 2012) in all embryonic CNS neurons caused Eve^+^ neuron loss (Figs. 5 D and S4). Conversely, *dsarm^4705^/dsarm^4621^* mutant embryos had more Eve^+^ neurons (Figs. 5 D and S4). Collectively, these data show that dSarm induces apoptosis and neuronal loss.

JNK is a common proapoptotic effector activated by p75^NTR^ and Sarm1 in mammals and Tolls in flies (Roux and Barker, 2002; Kim et al., 2007; Wu et al., 2015a). Thus, to ask whether dSarm induces apoptosis by activating JNK, we tested whether JNK knockdown could rescue apoptosis caused by *dsarm* overexpression. Indeed, overexpressing *dsarm* in all neurons together with *JNK-RNAi* decreased apoptosis compared with *Elav>EP3610* (Fig. 5 A). Thus, dSarm activates apoptosis via JNK. To further verify this, we asked whether MyD88 and dSarm affected activated pJNK^+^ cells in larval retinae. *MyD88^kra56^/Df(2R)BSC279* mutants had normal pJNK^+^ cell numbers, but overexpressing *dsarm* increased pJNK^+^ cell numbers (Fig. 5 E), and this increased further in a *MyD88^kra56^* mutant background (Fig. 5 E). This showed that dSarm activates apoptosis via JNK and antagonizes MyD88 function.

To test whether dSarm could inhibit MyD88 through direct physical interaction, we performed coimmunoprecipitations. S2 cells were cotransfected with *MyD88* tagged with V5 and *dsarm* tagged with HA. Precipitating MyD88 copurified dSarm, showing that dSarm and MyD88 interact physically (Fig. 5 F). Altogether, our data show that Sarm is an inhibitor of MyD88 and it induces apoptosis by antagonizing MyD88 and by activating JNK signaling.

But if neuronal apoptosis depends on dSarm, why did MyD88 induce apoptosis in pupae? We had shown that overexpression of *MyD88* increased neuron number, overexpression of *cactus* decreased Eve^+^ neuron number, and *MyD88* loss of function did not affect pJNK cell number, implying that NF-κΒ does not directly promote apoptosis. Importantly, apoptosis caused by *MyD88* overexpression in neurons was rescued by *JNK-RNAi* knockdown (Fig. 5 A), meaning that apoptosis downstream of MyD88 requires JNK. This suggests that MyD88 might induce apoptosis by up-regulating the expression of *JNK*, *weckle* (*wek*), or *dsarm*.

Our data had shown that Toll-6 can induce apoptosis and that it functions upstream of MyD88 to maintain neuronal survival, but MyD88 is inhibited by Sarm, which also induces apoptosis via JNK. So we asked whether Toll-6 and -7 could activate apoptosis by directly interacting with dSarm by using coimmunoprecipitations. We cotransfected S2 cells with *Toll-6–Flag* or *-7–Flag* and *dsarm-HA* and found that precipitating Toll-6 or -7 did not coprecipitate dSarm (Fig. 5 F). Thus, dSarm does not bind Toll-6 or -7, meaning that dSarm does not directly mediate the proapoptotic function of Toll-6.

Thus, our data show that Toll-6 functions upstream of dSarm and MyD88 to regulate neuronal death and survival, respectively (Fig. 5 G). But these data raise further questions: How can Toll-6 induce apoptosis if it does not bind dSarm? And why does Toll-6 promote cell survival in embryos and apoptosis in pupae?

### Proapoptotic Toll-6 signaling requires Wek

**Figure 6.**
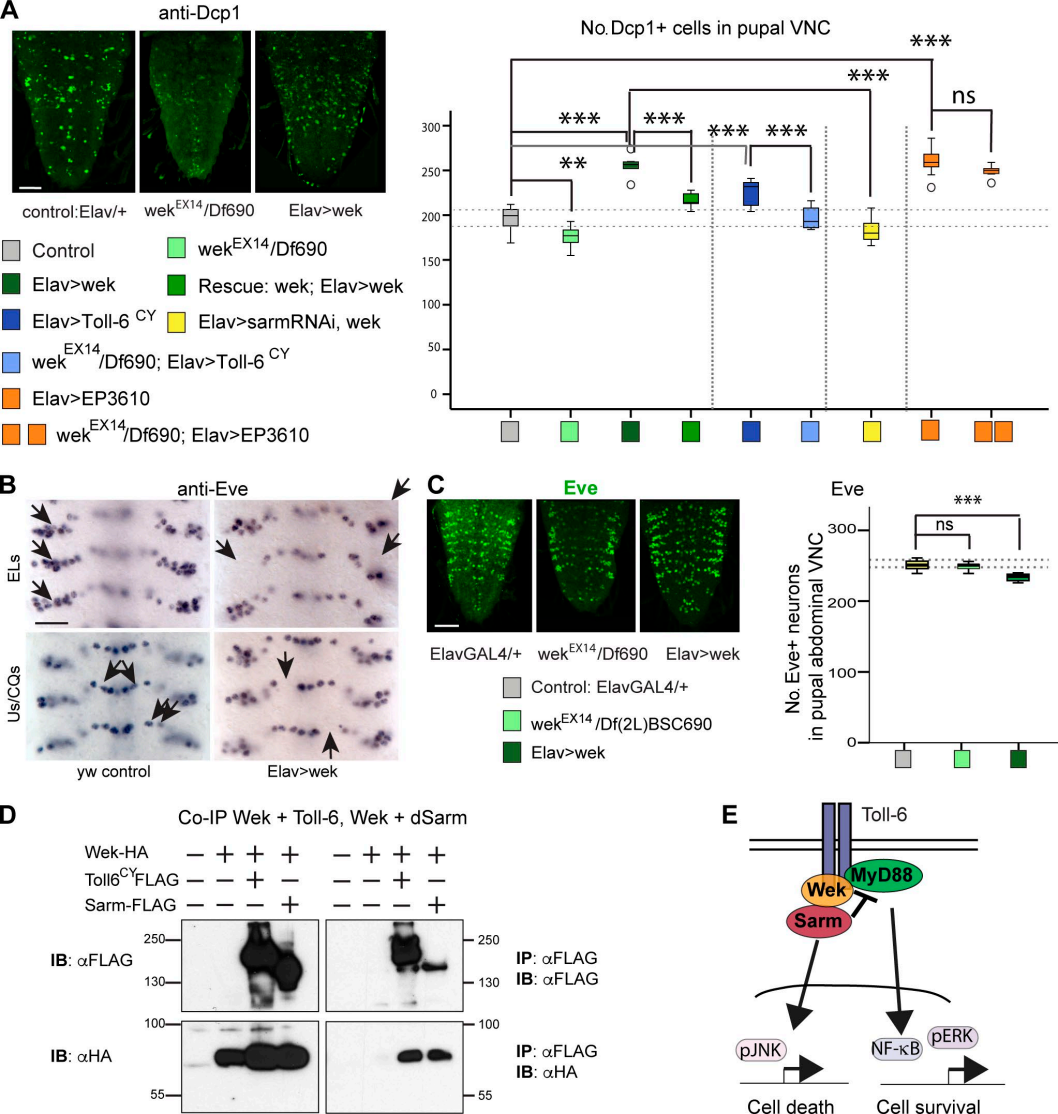
**Wek mediates the proapoptotic function of Toll-6 upstream of dSarm.** (A) Apoptotic cells in the white pupal VNCs visualized with anti-Dcp1 and quantified with DeadEasy. Box plot: Welch’s analysis of variance, P < 0.001, Bonferroni’s post-hoc test. *n* = 8–16. (B and C) Overexpression of *wek* in all neurons with *elav-GAL4* caused loss of Eve^+^ neurons in embryos (B; arrows indicate cells present in control and missing in *elav>wek*; quantification in Fig. S4) and pupae (C). Box plot: one-way analysis of variance, P < 0.001, Dunnett’s post-hoc test. Bars, 50 µm. *n* = 5–13. Asterisks on graphs indicate post-hoc multiple comparisons corrections: **, P < 0.01; ***, P < 0.001; ns, not significant. See Table S2. > indicates GAL4/UAS. (D) Coimmunoprecipitation from S2 cells showing that Wek binds Toll-6 and dSarm. IB, immunoblot; IP, immunoprecipitation. Molecular masses are given in kilodaltons. (E) Wek recruits dSarm and MyD88.

Our data suggest there might be another adaptor linking Toll-6 to dSarm to enable proapoptotic signaling. Wek is an adaptor downstream of Toll-1 that recruits MyD88 to form a signaling complex during embryonic development but not in innate immunity (Chen et al., 2006). To test whether proapoptotic Toll-6 signaling requires Wek, we measured apoptosis in the pupal VNC with anti-Dcp1 in loss- and gain-of-function genotypes. Apoptosis levels decreased in *wek^EX14^/Df(2L)BSC690* mutants compared with controls (Fig. 6 A). Conversely, overexpression of *wek* in neurons increased apoptosis (Fig. 6 A). These phenotypes were rescued by the overexpression of *wek* in neurons in a *wek* mutant background (Fig. 6 A). Thus, Wek can promote apoptosis in the CNS.

To test the relationship of Wek with Toll-6 and dSarm, we performed epistasis analyses. Loss of *wek* function rescued the increased apoptosis caused by the overexpression of *Toll6^CY^ (*Fig. 6 A), showing that Toll-6 requires Wek to induce apoptosis. Loss of *wek* function did not rescue the apoptosis caused by the overexpression of *dsarm*, meaning that dSarm functions downstream of Wek (Fig. 6 A). Furthermore, *dsarm* knockdown rescued the apoptosis caused by the overexpression of *wek*, showing that Wek induces apoptosis upstream of *dsarm* (Fig. 6 A). In embryos, overexpression of *wek* caused Eve^+^ neuron loss (Figs. 6 B and S4). In pupae, the number of Eve^+^ neurons did not change in *wek^EX14^/Df(2L)BSC690* mutants, but decreased upon *wek* overexpression (Fig. 6 C). Collectively, these data show that Wek can promote apoptosis and neuronal loss downstream of Toll-6 and upstream of dSarm.

To test whether Wek could bind Toll-6 and dSarm, we performed coimmunoprecipitations. We cotransfected S2 cells with *wek-HA* and *Toll-6–Flag* or *dsarm–Flag* and found that precipitating Toll-6 or dSarm also brought down Wek (Fig. 6 D). Thus, Wek can bind both Toll-6 and dSarm.

To conclude, Wek is required downstream of Toll-6 to induce neuronal apoptosis via dSarm (Fig. 6 E). But a question still remained: Why could Toll-6 promote cell survival in embryos and cell death in pupae?

### Adaptor profiles change in space and time

**Figure 7.**
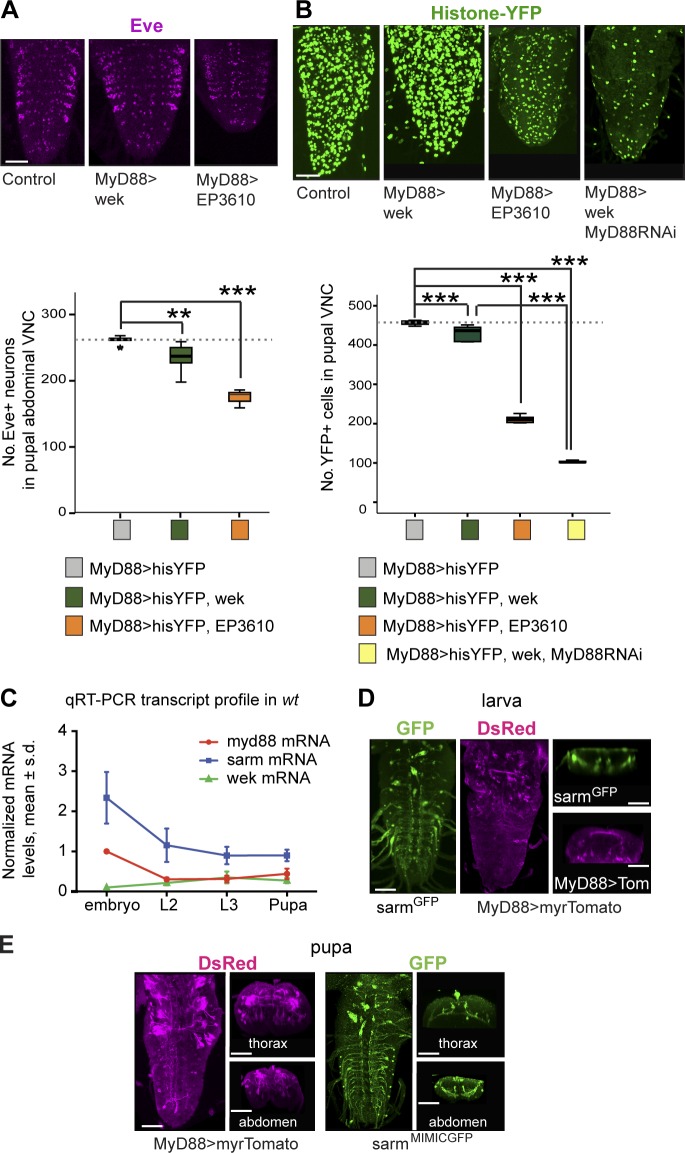
**Adaptors matter and change in space and time.** (A) Overexpression of *wek* or *dsarm (EP3610)* with *MyD88-GAL4* decreased Eve^+^ neuron numbers in pupal VNCs. Box plot (bottom): one-way analysis of variance, P < 0.001; Dunnett’s post-hoc test. *n* = 6–9. (B) *MyD88-*expressing cells visualized with *MyD88-GAL4^NP6394^* and nuclear *Histone-YFP* are lost in pupae by altering levels of adaptors. Box plot (bottom): Welch’s analysis of variance, P < 0.001, Bonferroni’s post-hoc test. *n* = 5–9. Dotted lines indicate medians of controls. (C) qRT-PCR showing a temporal profile of mRNA levels for *MyD88*, *dsarm*, and *wek* from whole embryos, L2 and L3 larval CNSs, and pupal CNSs, normalized to *MyD88* mRNA in embryos (three biological replicates; error bars represent means ± SD). *wt*, wild type; s.d., standard deviation. Asterisks on graphs indicate post-hoc multiple comparisons corrections: **, P < 0.01; ***, P < 0.001. See Table S2. > indicates GAL4/UAS. (D and E) Expression of *MyD88* visualized with *MyD88^NP6394^-GAL4>20×UAS-myr-td-tomato* and anti-DsRed and of *dsarm* visualized with *Ect4^MI08854^-GFP* and anti-GFP in the VNC of L3 larvae and pupae. Horizontal and transverse views are on the right for each. Bars, 50 µm.

Our data suggest that the relative levels of MyD88, dSarm, and Wek could determine neuronal life or death. Thus, we used *MyD88-GAL4* to ask how increasing the levels of Wek and Sarm relative to normal MyD88 levels would affect neurons. Overexpression of *wek* in MyD88^+^ cells decreased Eve^+^ neuron numbers in pupae compared with controls, and overexpression of *sarm (EP3610)* decreased Eve^+^ neurons further (Fig. 7 A). Using the nuclear reporter *Histone-YFP*, overexpression of *wek* reduced cell numbers in pupae, and overexpression of *dsarm* reduced cell numbers even further (Fig. 7 B). Remarkably, concomitant neuronal overexpression of *wek* with *MyD88* knockdown resulted in the most severe cell loss in pupal VNCs (Fig. 7 B). Because overexpression of *wek* alone had only a mild effect, this reveals that normally Wek is in a tug of war between dSarm and MyD88 signaling, that MyD88 and dSarm have antagonistic functions regulating cell numbers, and that Wek can engage both pathways downstream of Toll-6. Thus, relative levels of Wek, Sarm, and MyD88 determine cell survival or death downstream of Tolls.

Toll-6 maintains neuronal survival in embryos and can promote both neuronal survival and death in pupae, suggesting that its signaling adaptors change over time. To test this, we used real-time quantitative RT-PCR (qRT-PCR) and measured *MyD88*, *dsarm*, and *wek* transcript levels in whole stage 17 embryos and in the dissected CNS of second and third instar larvae (L2 and L3) and 1-d-old pupae. *MyD88* mRNA levels were high in embryos, decreased in L2 CNS, and increased again between L3 and white pupae (Fig. 7 C). Relative to MyD88 transcripts, *dsarm* mRNA levels were high in embryos, decreasing thereafter (Fig. 7 C), and *wek* mRNA levels were virtually absent in embryos and increased from L2 on (Fig. 7 C). *wek* expression was consistently lower than that of *dsarm* and equal to *MyD88* from L2 onward (Fig. 7 C). The low levels of Wek in embryos suggest that in the embryonic CNS, Toll-6 can bind MyD88 to activate cell survival, but because there is no Wek, it cannot activate the dSarm proapoptotic pathway. In the pupa, in the presence of Wek, Toll-6 can activate either cell survival via MyD88 or cell death via dSarm. Thus, the temporal regulation of *wek* expression explains the different outcomes of Toll-6 function over time.

To visualize whether the spatial distribution of MyD88 and dSarm may also change, we used a *dsarm^MIMIC^-GFP* insertion and *MyD88-GAL4^NP6394^* to drive the expression of membrane-tethered *10×UAS-myr-td-Tomato* and anti-DsRed antibodies. Both were widely expressed throughout the embryonic CNS neuropile (Figs. 4 and 5), widespread in larvae (Fig. 7 D), and more restricted in pupae (Fig. 7 E). In pupae, *MyD88>myr-td-Tomato* was distributed throughout the VNC, but prominently in thoracic interneurons potentially linked to the motor circuitry (Fig. 7 E). dSarm^MIMIC^-GFP was distributed throughout the VNC but prominently in ventral projections, apparently sensory circuits (Fig. 7 E). These distinct patterns suggest that after cell number regulation, neural circuits acquire a characteristic composition of Toll signaling adaptors.

### Mammalian NTs can induce signaling from mammalian TLRs

To test whether the link between NTs and Toll receptors might also occur in mammals, we performed signaling assays with TLR2 and TLR4, which are cell membrane receptors present in the mammalian brain, and TLR5, an intracellular receptor (Gay et al., 2014). HEK293T cells were transfected with TLR2, 4, and 5 and an NF-κB luciferase reporter, and signaling was measured after stimulation with increasing concentrations of mature BDNF or NGF (Fig. S5). Whereas there was no effect upon stimulation of TLR2 or TLR5 with either NGF or BDNF, both ligands induced signaling in cells transfected with TLR4 (Fig. S5). Furthermore, treatment with NGF or BDNF altered the response of TLR2, 4, and 5 to stimulation with their canonical innate immunity ligands (Fig. S5). This means that mammalian NTs can influence mammalian TLR signaling.

## Discussion

DNTs and Tolls regulate cell number plasticity by promoting both cell survival and death in the *Drosophila* CNS through a three-tier mechanism.

**Figure 8.**
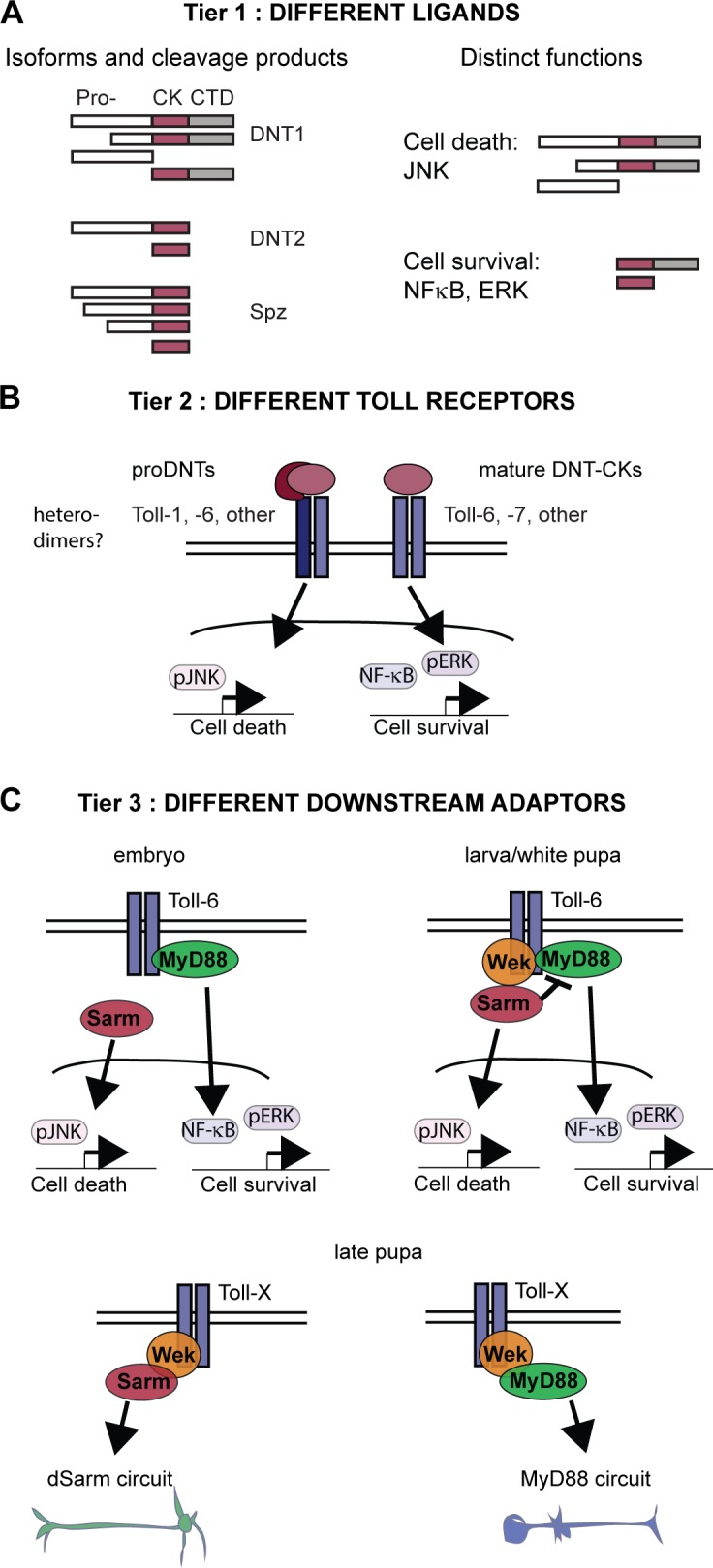
**Three-tier regulation of cell number plasticity by DNTs and Tolls.** (A) Tier 1: different ligand forms result from cleavage by furin proteases and isoforms and can lead to different cellular outcomes. Pro-, prodomain. (B) Tier 2: different Tolls can lead to different outcomes. (C) Tier 3: different adaptors downstream of Tolls drive alternative cellular outcomes. Adaptor expression changes in time and space. In embryos, Wek levels are low, and dSarm and MyD88 have independent functions. As Wek levels rise, it recruits dSarm and MyD88, and dSarm inhibits MyD88. Toll-6 promotes cell survival via MyD88 in the embryonic CNS, and with Wek it can also induce apoptosis in pupa. Surviving cells segregate into potentially overlapping but distinct neural circuits.

In the first tier, each DNT has unique features conducive to distinctive functions (Fig. 8 A). Spz, DNT1, and DNT2 share with the mammalian NTs the unequivocal structure of the CK domain unique to this protein family. However, DNT1, DNT2, and Spz have distinct prodomain features and are processed differently, leading to distinct cellular outcomes (Fig. 8 B). Spz is only secreted full length and cleaved by serine proteases (Hoffmann and Reichhart, 2002). DNT1 and 2 are cleaved intracellularly by conserved furins. In cell culture, DNT1 was predominantly secreted with a truncated prodomain (pro-DNT1), whereas DNT2 was secreted mature. In vivo*,* both pro- and mature DNTs were produced from neurons. Interestingly, DNT1 also has an isoform lacking the CK domain (Zhu et al., 2008), and Spz has multiple isoforms with truncated prodomains (DeLotto et al., 2001). Thus, in vivo*,* whether DNT1 and 2 are secreted full length or cleaved and whether Spz is activated will depend on the proteases that each cell type may express. Pro-DNT1 activates apoptotic JNK signaling, whereas mature DNT1 and 2 activate the prosurvival NF-κB (Dorsal and Dif) and ERK signaling pathways. Mature Spz does not activate ERK. This first tier is evolutionarily conserved, as mammalian pro-NTs can promote cell death, whereas furin-cleaved mature NTs promote cell survival (Lu et al., 2005). NF-κB, JNK, and ERK are downstream targets shared with the mammalian NTs, downstream of p75^NTR^ (NF-κB and JNK) and Trks (ERK), to regulate neuronal survival and death (Roux and Barker, 2002; Lu et al., 2005; Minichiello, 2009). Thus, whether a cell lives or dies will depend on the available proteases, the ligand type, and the ligand cleavage product it receives (Fig. 8 A).

In a second tier, we showed that the specific Toll family receptor activated by a DNT matters (Fig. 8 B). Toll-6 and -7 could maintain neuronal survival, whereas Toll-1 had a predominant proapoptotic effect. Because there are nine Tolls in *Drosophila*, some Tolls could have prosurvival functions, whereas others could have proapoptotic functions. Different Tolls also lead to different cellular outcomes in immunity and development (Tauszig et al., 2000; Yagi et al., 2010; McIlroy et al., 2013; Meyer et al., 2014; Paré et al., 2014). Thus, the life or death of a neuron will depend on the Toll or combination of Tolls it expresses (Fig. 8 B). We also showed that binding of Spz to Toll-1 is most likely unique, but DNT1 and 2 bind Toll-6 and -7 promiscuously, and, additionally, we showed that DNT1 and 2 with Toll-6 and -7 activate NF-κB and ERK, whereas pro-DNT1 activates JNK. This suggests that ligand prodomains might alter the affinity for Toll receptors and/or facilitate the formation of heterodimers between different Tolls and/or with other coreceptors to induce cell death. A “DNT–Toll code” may regulate neuronal numbers.

In a third tier, available downstream adaptors determine the outcome between cell survival and death (Fig. 8 C). Toll-6 and -7 activate cell survival by binding MyD88 and activating NF-κB and ERK (whether ERK activation depends on MyD88 is not known), and Toll-6 can activate cell death via Wek, dSarm, and JNK signaling. We have shown that Toll-6 binds MyD88 and Wek, which binds dSarm, and that dSarm binds MyD88 and promotes apoptosis by inhibiting MyD88 and activating JNK. Wek also binds MyD88 and Toll-1 (Chen et al., 2006). So, evidence suggests that Wek recruits MyD88 and dSarm downstream of Tolls (Fig. 8 C). Because Toll-6 binds both MyD88 and Wek and Wek binds both MyD88 and dSarm, Wek functions like a hinge downstream of Toll-6 to facilitate signaling via MyD88 or dSarm, resulting in alternative outcomes. Remarkably, adaptor expression profiles change over time, switching the response to Toll-6 from cell survival to cell death. In the embryo, when both MyD88 and dSarm are abundant, there is virtually no Wek, and Toll-6 can only bind MyD88 to promote cell survival (Fig. 8 C). As Wek levels rise, Toll-6 signaling can also induce cell death. If the Wek-Sarm-JNK route prevails, Toll-6 induces apoptosis; if the Wek–MyD88–NF-κB route prevails, Toll-6 signaling induces cell survival (Fig. 8 C).

Thus, the cellular outcome downstream of DNTs and Tolls is context and time dependent. Whether a cell survives or dies downstream of DNTs and Tolls will depend on which proteases are expressed nearby, which ligand it receives and in which form, which Toll or combination of Tolls it expresses, and which adaptors are available for signaling (Fig. 8).

How adaptor profiles come about or change is not understood. A neuronal type may be born with a specific adaptor gene expression profile, or Toll receptor activation may influence their expression. In fact, MyD88 reinforces its own signaling pathway, as Toll-6 and -7 up-regulate Dorsal, Dif, and Cactus protein levels (McIlroy et al., 2013) and TLR activation increases Sarm levels (O’Neill and Bowie, 2007). We showed that apoptosis caused by MyD88 excess depends on JNK signaling. Because JNK functions downstream of Wek and dSarm, this suggests that MyD88, presumably via NF-κB, can activate the expression of *JNK*, *wek*, or *dsarm*. By positively regulating *wek* expression, MyD88 and dSarm could establish positive feedback loops reinforcing their alternative pathways (Fig. 8 C, bottom). Because dSarm inhibits MyD88, mutual regulation between them could drive negative feedback. Positive and negative feedback loops underlie pattern formation and structural homeostasis and could regulate neuronal number in the CNS as well. Whether cell-autonomous or -nonautonomous mechanisms result in the diversification of adaptor profiles, either in time or cell type, remains to be investigated.

Either way, over time the Toll adaptors segregate to distinct neural circuits, where they exert further functions in the CNS (Fig. 8 C). Toll-1, -6, and -8 regulate synaptogenesis and structural synaptic plasticity (Halfon et al., 1995; Ballard et al., 2014; McLaughlin et al., 2016). Sarm regulates neurite degeneration, and in the worm, it functions at the synapse to determine neuronal identity (Chuang and Bargmann, 2005; Osterloh et al., 2012). The reporters we used revealed a potential segregation of MyD88 to the motor circuit and dSarm to the sensory circuit, but this is unlikely to reflect the endogenous complexity of Toll-signaling circuitry, as *dsarm^MIMIC^*^−^ has a GFP insertion into one of eight potential isoforms, and *dsarm* also functions in the motor system (McLaughlin et al., 2016). Importantly, cell death in the normal CNS occurs mostly in late embryogenesis and in pupae, coinciding with neural circuit formation and remodeling, when neuronal number is actively regulated. Thus, the link by DNTs and Tolls from cell number to circuitry offers a complex matrix of possible ways to regulate structural plasticity in the CNS.

We have uncovered remarkable similarities between *Drosophila* Toll-6 and mammalian TLR signaling involving MyD88 and Sarm. All TLRs except TLR3 signal via MyD88 and activate NF-κB (Gay and Gangloff, 2007; Gay et al., 2014). Neuronal apoptosis downstream of TLRs is independent of NF-κB and instead depends on TRIF and Sarm1 (Kaiser and Offermann, 2005; Ma et al., 2006; Kim et al., 2007; Mukherjee et al., 2013). Sarm1 is a negative regulator of TLR signaling, an inhibitor of MyD88 and TRIF (Carty et al., 2006). *sarm1* is expressed in neurons, where it activates JNK and promotes apoptosis (Kim et al., 2007; Osterloh et al., 2012; Mukherjee et al., 2013). However, the endogenous ligands for TLRs in the normal undamaged brains are not known. Our preliminary analysis has revealed the intriguing possibility that NTs either can bind TLRs or induce interactions between Trks, p75^NTR^, and TLRs. It is compelling to find out whether TLRs regulate structural plasticity in the mammalian brain in concert with NTs.

To conclude, DNTs with Tolls constitute a novel molecular system for structural plasticity in the *Drosophila* CNS. This could be a general mechanism to be found also in the mammalian brain and in other contexts as well, such as epithelial cell competition and regeneration, and altered in cancer and neurodegeneration.

## Materials and methods

### Genetics

#### Mutant and reporter stocks

Control stocks were *yw* and/or outcrosses of *yw*, as most transgenic flies were in a *w*^−^ background. *MyD88^kra56^* is an ethyl methanesulfonate (EMS)–induced hypomorphic allele (a gift from B. Moussian; Charatsi et al., 2003), and *wek^EX14^* is an excision loss-of-function allele (a gift from J.L. Imler, Centre National de la Recherche Scientifique, Strasbourg, France; Chen et al., 2006). *dsarm^4705^* and *dsarm^4621^* are loss-of-function alleles of *dsarm* (a gift from Marc Freeman, University of Massachusetts Medical School, Worcester, MA). The deficiency *Df(2R)BSC279* lacks the *MyD88* locus and *Df(2L)BSC690* lacks the *wek* locus, respectively. *Dorsal-GFP* (*w^1118^;PBac{dl-GFP.FLAG}VK00033/TM3, Sb^1^)* and *Dif-GFP (w^1118^;Pbac{Dif-GFP.FPTB}VK00033)* are both GFP exon trap lines. *Ect4^MI00854^* and *sarm* are synonyms for the same gene, and *Ect4^MIMIC-GFP^ (yw;MiMicECT4[MI08854])* is a MIMIC insertion bearing GFP into the Ect4 locus. Stocks were balanced using CyOlacZ and TM6BlacZ, to identify mutant embryos, or SM6aTM6B balancers carrying Tb^−^, to identify mutant larvae and pupae. Double and triple mutants and other stocks were generated by conventional genetics.

#### Overexpression in vivo

We used the following GAL4 drivers: (a) *w;; elav-GAL4* for all neurons; (b) *w; GMR-GAL4* for the retina (a gift from Matthew Freeman, University of Oxford, Oxford, England, UK); (c) *w; RG-GAL4* drives expression in the ring gland and in a small neuronal cluster in the optic lobes; and (d) *w; MyD88-GAL4: yw;P{GawB}MyD88^NP6394^/Cyo, P{UAS-lacZ.UW14}UW14* (Bloomington Drosophila Stock Center). These were crossed to (a) the membrane-tethered reporter *w;; 10×UAS-myr-td-Tomato* (a gift from B.D. Pfeiffer, University of Texas Southwestern, Dallas, TX); (b) activated forms of Tolls *w;;UASToll-6^CY^* and *w;;UASToll-7^CY^* (McIlroy et al., 2013) and UASToll-1^10b^ (a gift from J.M. Reichhart, University of Strasbourg, Strasbourg, France); (c) *w; UAS-MyD88-FL* (a gift from J. Kagan, Harvard Medical School, Boston, MA); (d) *w; UAS-dsarm* (a gift from Marc Freeman), which drives expression of the *dsarm* cDNA (Osterloh et al., 2012), and *w^1118^;P{EP}EP3610/TM6B,Tb^1^*, which drives expression of all *Ect4 (dsarm)* isoforms (Bloomington Drosophila Stock Center; *Ect4* and *sarm* are synonyms for the same gene); (e) *UAS-wek-HA; UAS-cactus-HA* (FlyORF); or (f) *w[11]; UAS-JNK-RNAi [P(GD10555*) (VDRC34138) and *UAS-dsarmRNAi; UAS-MyD88-RNAi* (VDRC32396; Vienna Drosophila Research Center).

### Structural modeling of DNTs and comparison to mammalian NTs

DNT1 and 2 were modeled on their closest structural homologue, Spz, using Modeller software (Webb and Sali, 2014), which builds ab initio the loops that were not observed crystallographically in Spz. The same method was used to complete the 3D model of Spz. The structure of the BDNF protomer is known in the context of heterodimerization with either NT3 (Robinson et al., 1995) or NT4 (Robinson et al., 1999). We generated a 3D model of the BDNF homodimer based on these heterodimers by substituting the NT with BDNF and performing energy minimization in Modeller (Webb and Sali, 2014). Protein sequences were analyzed by Clustal Omega (Sievers et al., 2011) and Tcoffee (Notredame et al., 2000). Figures were generated in PyMol (DeLano Scientific) using the lowest energy models with least clashes and best geometry according to Verify3D (Bowie et al., 1991) and MolProbity (Chen et al., 2010), respectively.

### Bioinformatics and sequence analysis

#### Analysis of prodomain

Analysis was performed using PSIPRED, a secondary structure prediction program.

#### Identification of conserved furin sites

Potential furin cleavage sites in DNT1 and 2 were identified by the PiTou prediction tool (Tian et al., 2012). To test the predicted cleavage sites, mutant DNT1 and 2 constructs were generated by site-directed mutagenesis (see the Site-directed mutagenesis section). S2 cells were transfected with full-length, truncated, or mutant forms of DNT1 or 2 cloned into *pAct5c-3×HA* expression vector (see the Cell culture, transfection, stimulation . . . section). After transfection, cells were separated from culture media and lysed in NP-40 buffer (50 mM Tris-HCl, pH 8.0, 150 mM NaCl, and 1% Igepal CA-630). HA-tagged proteins in cell lysates and culture media were detected by anti-HA antibody using standard Western blots.

#### Primer design

Primers were designed using the public resource Primer3Plus. For site-directed mutagenesis, primers were designed using the QuickChange Primer Design online tool (Agilent Technologies). For qRT-PCR to detect which Toll receptors were expressed in S2 cells, Primer-BLAST was used to design specific primers.

### Molecular biology

#### Generation of fusion constructs

Full-length or truncated cDNAs of DNT1 and 2 were cloned into an expression vector using a standard Gateway procedure, inserting them first into *pDONR* and subsequently into *pAct5c-3×HA* to generate the following constructs: *pAct5c-DNT1-FL-3×HA*; *pAct5c-DNT1 (Sp* + *CK* + *CTD)* − *3×HA*; *pAct5c-DNT2-FL-3×HA*; and *pAct5c-DNT2 (Sp* + *CK)* − *3×HA.* Cloning to generate HA-tagged *dsarm* was also performed using the Gateway system. *dsarm* cDNA was amplified from a *pUAST-dsarm* plasmid (a gift from Marc Freeman) and then was subcloned first into pDONR and subsequently into the destination vector, resulting in *pAct5c-dsarm-3×HA*. *UAS-DNT1-FL-GFP* and *UAS-DNT2-FL-GFP* were tagged at the C terminus with GFP by cloning: *DNT1-FL-GFP* was cloned into pUASt using conventional ligation and transgenesis, and *DNT2-FL-GFP* was cloned by Gateway cloning into *pUAS-GW-GFP* followed by conventional transgenesis, using *white* as the selection marker. For all primers, see Table S1.

#### Generation of *MyD88^cr2.8^* mutant allele by CRISPR/Cas9

A *MyD88* CRISPR mutant allele was created by designing a guide RNA targeting exon 1 of MyD88 using CRISPR software (Massachusetts Institute of Technology) with the primers MyD88 BbSI sense, 5′-GTCGCCGAGGGAGTTATGGACTCC-3′, and antisense, 5′-AAACGGAGTCCATAACTCCCTCGG-3′, cloned in to the BbsI site of the pCFD3 U6.3 vector and verified by sequencing. Transgenic flies bearing U6.3 MyD88 guide RNA were generated by φC31 transgenesis (injections by BestGene Inc). Flies bearing the guide RNA (*yscv;;U6.3MyD88gRNA attp2/TM3(sb)*) were crossed to flies carrying Cas9 driven by the nanos promoter *ym{nosCas9}ZH2A*. Independent balanced stocks were established from F1 males (*w;MyD88^CRISPR^/CYO*) and sequenced. *MyD88^cr2.8^* bears a 7-bp deletion that causes a frameshift at amino acid 64 and a premature stop codon at amino acid 94. This corresponds to the start of the death domain (amino acids 90–172). This allele lacks the death and Toll–interleukin receptor domains and is therefore a null allele. The sequence of the lesion and the amino acid sequence are given in Table 1. 

**Table 1.** Lesion and amino acid sequences used to generate the *MyD88^CR2.8^* allele

Allele	Sequence (5′–3′)
**Lesion**	
MyD88^WT^	GTCAGTTATCGGCGTTATCGCACCGCTGGCATGGTGGTG**GCCGAGGGAGTTATGGACTCC**
MyD88^CR2.8^	GTCAGTTATCGGCGTTATCGCACCGCTGGCATGGTGGTGGCCGAGGGAGTTATG
MyD88^WT^	GGGTCGGGATCGGGCACGGGAACGGGCTTGGGGCACTTCAACGAGACCCCATTATCCGCA
MyD88^CR2.8^	GGTCGGGATCGGGCACGGGAACGGGCTTGGGGCACTTCAACGAGACCCCATTATCCGCA
**Amino acid**	
MyD88^WT^	MRPRFVCHQQHSVAHSHYQPHSHFHHHTHRHPNPPHHHHIYGATDVSYRRYRTAGMVV**AE**
MyD88^CR2.8^	MRPRFVCHQQHSVAHSHYQPHSHFHHHTHRHPNPPHHHHIYGATDVSYRRYRTAGMVV**AE**
MyD88^WT^	**GVMD**SGSGSGTGTGL------GHFNETPLSALGIETRTQLSRMLNRKKVLRSEEGYQRDW
MyD88^CR2.8^	**GVMG**RDRARERAWGTSTRPHYPHWASRPAPSCPAC**STOP**

Guide RNA sequences are given in bold.

#### RT-PCR

RT-PCR was performed to see which Toll receptors were expressed in S2 cells. Total RNA was isolated from S2 cells by TRIzol (Ambion) reagent following a standard protocol. Reverse transcription was performed by using the GoScript system (Promega). The standard PCR reaction was performed to amplify Toll receptor cDNA fragments using *Taq* DNA polymerase (Invitrogen). For a list of primers, see Table S1.

#### Site-directed mutagenesis

One or more point mutations were generated in *pAct5c-DNT1-FL-3×HA* and *pAct5c-DNT2-FL-3×HA* fusion constructs by site-directed mutagenesis according to Wang and Malcolm (1999). The following mutant expression clones were used for S2 cell transfection: *pAct5c-DNT1-FL-R499G-3×HA*, *pAct5c-DNT1-FL-R283/499G-3×HA*, *pAct5c-DNT2-FL-R284G*, and *pAct5c-DNT2-FL-R214/221/284G-3×HA.* For primers, see Table S1.

#### qRT-PCR

From 2 h–staged egg collections at 25°C, whole dechorionated embryos were harvested 20 h after egg laying (AEL), and the CNS was dissected from L2 larvae at 48 h AEL, L3 larvae at 96 h AEL, and pupae 0–12 h after puparium formation. Samples were then placed immediately into TRI reagent (AM9738; Ambion) and frozen at −80°C. Total RNA was extracted from 20 embryos or 20 dissected larval or pupal CNSs using TRl and following the manufacturer’s instructions. cDNA was synthesized from 200 ng of total RNA using the GOScript reverse transcription system (A5001; Promega) using random primers and then diluted threefold for quantitative PCR reactions, and 2 µl was used per reaction. “No reverse transcription” controls were run alongside cDNA reactions. Transcript levels were determined in triplicate for each sample using SensiFAST Hi-ROX SYBR green (BIO-92020; Bioline) run on a sequence detection system (ABI PRISM 7000; Thermo Fisher Scientific). The reference gene was *RpL32*, as it remained constant over the course of development. Primers used are given in Table S1.

To obtain fold change values by using the 2^−ΔΔCt^ method (Livak and Schmittgen, 2001) for the developmental profiles of *MyD88*, *dsarm*, and *wek*, the Ct value of *Rpl32* was subtracted from the Ct value of each gene and developmental time point to obtain ΔCt. All values were then normalized to the calibrator, which. for this set of experiments, was *MyD88* mRNA at embryo (ΔΔCt). Three independent biological replicates were performed per experiment, and the mean ± SD is provided in Fig. 5 B.

### Cell culture

#### Cell culture, transfection, stimulation, and subcellular fractionation

S2 cells were maintained at 27°C in InsectXpress medium (Lonza) supplemented with 10% heat-inactivated FBS and 1% penicillin–streptomycin–glutamine (Gibco). Transfection reagent (TransIT2020; Mirus) was used to express target proteins in S2 cells.

To stimulate S2 cells with mature DNT2-CK, S2 cells were transfected with *pAct5c-Toll-6-3×HA* or *pAct5c-Toll-7-3×HA* and were grown overnight in a 6-well plate (2 × 10^6^ cells/well). Cells were serum starved for at least 6 h and then were treated with purified DNT2-CK (50 nM) for 5–60 min.

To separate nuclear and cytoplasmic fractions, cells were pelleted and washed in ice-cold PBS at 500 *g* for 5 min at 4°C. The cells were lysed in ice-cold harvest buffer (10 mM Hepes, pH 7.9, 50 mM NaCl, 0.5 M sucrose, 0.1 mM EDTA, 0.5% TritonX-100, 1 mM DTT supplemented with a protease inhibitor cocktail [Thermo Fisher Scientific] and 5 mM NaF, and 2 mM Na_3_VO_4_) for 5 min on ice. Lysate was spun at 800 *g* for 10 min at 4°C. Supernatant was treated as cytoplasmic/membrane and pellet was treated as nuclear fraction. The cytoplasmic/membrane fraction was transferred in an empty tube and subsequently purified by centrifugation at 14,000 *g* for 10 min at 4°C. The nuclear pellet was resuspended in buffer A (10 mM Hepes, pH 7.9, 10 mM KCl, 0.1 mM EDTA, 0.1 mM EGTA, 1 mM DTT supplemented with protease inhibitor cocktail and 5 mM NaF, and 2 mM Na_3_VO_4_) and spun at 800 *g* for 10 min at 4°C. Supernatant was discarded and the pellet was resuspended in buffer C (10 mM Hepes, pH 7.9, 500 mM NaCl, 0.1 mM EDTA, 0.1 mM EGTA, 0.1% NP-40, 1 mM DTT supplemented with a protease inhibitor cocktail and 5 mM NaF, and 2 mM Na_3_VO_4_) and incubated on ice for 30 min. Nuclear fraction was purified by centrifugation at 14,000 *g* for 10 min at 4°C.

To analyze total cell lysate, S2 cells were pelleted and washed in ice-cold PBS and then lysed in radioimmunoprecipitation assay buffer (10 mM Tris-HCl, pH 8.0, 150 mM NaCl, 1 mM EDTA, 1% Triton X-100, 0.1% SDS, 0.1% sodium deoxycholate supplemented with a protease inhibitor cocktail and 5 mM NaF, and 2 mM Na_3_VO_4_). Total cell lysate or subcellular fractions were analyzed by SDS-PAGE followed by Western blotting using standard procedures.

#### Coimmunoprecipitations from cotransfected S2 cells

Coimmunoprecipitations from S2 cells were performed as previously described (McIlroy et al., 2013). S2 cells were transfected with the following combinations of plasmids: (a) *pAct5c-Toll-6-3×HA* and *pAct5c- Pro-TEV6HisV5-DNT1-CK-CTD*; *pAct5c-Toll-7-3×HA* and *pAct5c-Pro-TEV6HisV5-DNT2-CK*; (b) *pAct5c-MyD88-V5* and *pAct5c-Toll-6-3×Flag* or *pAct5c-Toll-6^CY^-3×Flag*, *pAct5c-Toll-7-3×Flag*, or *pAct5c-Toll-7^CY^-3×Flag*; (c) *pAct5c-dsarm-3×HA* and *pAct5c-MyD88-V5*; *pAct5c-dsarm-3×HA* and *pAct5c-Toll-6^CY^-3×Flag* or *pAct5c-Toll-7^CY^-3×Flag*; and (d) *pAct5c-Wek-3×HA* and *pAct5c-Toll6^CY^-3×Flag* or *pAct5c-dsarm-3×Flag*. *pAct5c-MyD88-V5* plasmid was a gift from S. Wasserman (University of San Diego, San Diego, CA); *pAct5c-Wek-3×HA* was a gift from J.L. Imler (Institut de Biologie Moléculaire et Cellulaire, Strasbourg, France). Cells were collected 48 h after transfection and lysed in NP-40 buffer or in Flag affinity chromatography buffer (50 mM Hepes, pH 7.5, 80 mM KCl, 5 mM MgCl_2_, 2 mM EGTA, and 0.2% Triton X-100) supplemented with protease inhibitor cocktail. Immunoprecipitations from lysates were performed using mouse anti-V5 antibody in combination with protein-A/G magnetic beads (Thermo Fisher Scientific) or anti-Flag antibody-conjugated agarose or magnetic beads (Sigma-Aldrich). Proteins were analyzed by SDS-PAGE and Western blotting as described in the Western blots section.

#### Luciferase reporter assay in mammalian cells

HEK293 cells were seeded at 10^5^ cells/well in a 96-well plate 36 h before transfection with jetPEI (Polyplus). NF-κB–dependent gene expression was determined using a luciferase reporter construct concomitantly with indicated TLR vectors. The Renilla luciferase-thymidine kinase–encoding plasmid (pRL-TK) was used to normalize for transfection efficiency, and pcDNA3.1 empty vector was used to maintain constant DNA. Cells were stimulated in a dose-dependent manner using neurotrophic agents hNGF-β (H9666; Sigma-Aldrich), hBDNF (R&D Systems), or mNGF-7S (N0513; Sigma-Aldrich). Transfected cells were lysed using Passive lysis buffer and assayed for luciferase and Renilla activity using luciferase assay reagent (Promega). Luminescence readings were corrected for Renilla activity and expressed as fold increases over unstimulated control values. Data are presented as means ± SEM of one of three independent experiments. Statistical analysis was performed using a two-way analysis of variance where we compared TLR signaling upon stimulation with varying concentrations of NTs or upon stimulation with both canonical innate immunity ligands and NTs.

### Immunostainings

#### In vivo immunostainings in the larval and pupal CNS

Dissections, fixations, and immunostainings were performed following standard procedures, except that for stainings to detect apoptosis in the pupal CNS, only pupae within the first 10 min of puparium formation were used in order to minimize biological variability in apoptosis levels over time. Primary antibodies used were rabbit anti-GFP (1:500 in larvae and pupae, 1:1,000 in embryos; A11122; Invitrogen), rabbit anti-DsRed (1:100; 632496; Takara Bio Inc.), rabbit anti-βgal (1:5,000; Cappel), mouse anti-Eve (1:5–1:10; 2B8; Developmental Studies Hybridoma Bank), mouse anti-Eve (1:20; 3C10; Developmental Studies Hybridoma bank), mouse anti-pERK (1:500 in retina and 1:100 in optic lobe; 9106; Cell Signaling Technology), mouse anti-Repo (1:250; 8D12; Developmental Studies Hybridoma Bank), rabbit anti-pJNK (1:200; V7931; Promega), rabbit anti-Myd88 (1:250; a gift from S. Wasserman), and rabbit anti-Dcp1 (cleaved *Drosophila* Dcp1 [Asp216]; 1:500; 9578S; Cell Signaling Technology). Secondary antibodies were directly conjugated Alexa Fluor 488, 546, and 647 (1:250, Molecular Probes) or biotinylated mouse or rabbit (1:300) followed by avidin amplification using the Vectastain ABC Elite kit (Vector Laboratories) or the Tyramide Signal Amplification kit (T20922; Thermo Fisher Scientific), using the manufacturer’s instructions. For sample sizes, see Table S2.

#### Western blots

Western blotting was performed according to standard procedures. Primary antibodies used were mouse anti-V5 (1:5,000; R960-25; Invitrogen), rabbit anti-Flag (1:2,000; F7425; Sigma-Aldrich), mouse anti–histone-H1 (1:10,000; 05-629; EMD Millipore), mouse antitubulin (1:10,000; T9026; Sigma-Aldrich), chicken anti-HA (1:2,000 and 1:5,000; ET-HA100; Aves Lab), mouse anti-HA (12CA5; 1:2,000; 11 583 816 001; Roche), mouse anti-Dorsal (7A4; 1:500; Developmental Studies Hybridoma Bank), mouse anti-Cactus (3H1; 1:500; Developmental Studies Hybridoma Bank), and rabbit anti-Dif (1:500; a gift from D. Ferrandon, University of Strasbourg, Strasbourg, France). Secondary antibodies used were anti-mouse HRP (1:5,000; PI-2000; Vector Laboratories), anti-rabbit HRP (1:5,000; PI-1000; Vector Laboratories), and anti-chicken HRP (1:10,000; 703-035-155; Jackson ImmunoResearch Laboratories, Inc.).

### Microscopy and imaging

#### Imaging

For microscopy, samples were mounted either in 70% glycerol and 30% PBTriton (larval and pupal fluorescent CNS and nonfluorescent embryos) or in Vectashield (H-1000; Vector Laboratories; fluorescent embryonic CNS). Wide-field microscopy was performed with a microscope (Axioplan 2; ZEISS) and a 63× objective; images were taken under Nomarski optics with an AxioCam color camera and Zen software (ZEISS). Fluorescent microscopy was performed using secondary antibodies directly conjugated to Alexa Fluor 488, 546, and 647. Laser-scanning confocal microscopy was performed at room temperature using a spectral confocal microscope (SP2 AOBS; Leica Microsystems) and a 40× or 63× lens at 512 × 512– or 1,024 × 1,024–pixel resolution and with 0.5- or 1-µm steps or a laser-scanning microscope (LSM 710; ZEISS) with a 25× oil lens at 512 × 512–pixel resolution and with 1-µm steps. Confocal image acquisition was performed with Leica Microsystems or ZEISS software as per the system. Each confocal stack comprised 100–300 images, which were processed as follows: (a) for image data, ImageJ (National Institutes of Health) was used to view the entire stack of images, carry out horizontal and transverse projections, and rotate images; occasionally, a median or “dust and scratches” filter was applied to a projection image over the whole image. Photoshop (Adobe) was used to adjust levels, rotate and crop images, and adjust image size to 300 dpi. Illustrator (Adobe) was used to compile figure plates. (b) For quantitative data (e.g., number of Dcp1^+^, Eve^+^, or YFP^+^ cells), we used the ImageJ plugins DeadEasy Larval Glia (which counts nuclear stains) and DeadEasy Caspase for Larvae (for apoptotic cells) as previously described and validated (Forero et al., 2009, 2012; Kato et al., 2011). DeadEasy analyzes the entire stacks of images in 3D, identifies cells based on pixel intensity and 3D volume, and counts cells automatically in an entire CNS in 3D in less than a minute.

#### Quantitative data analysis

Penetrance is the frequency with which a phenotype is manifested within a population, and expressivity is the severity of the phenotype. Eve^+^ cells in embryos analyzed under wide-field Nomarski optics were counted manually under an Axioplan 2 microscope and a 63× objective. Fluorescent pJNK^+^ cells in the retina were counted manually within the stacks of confocal sections using ImageJ and the Cell Counter macro.

Dcp1^+^ apoptotic cells from the entire VNC of the CNS were counted automatically using DeadEasy Caspase for Larvae(Forero et al., 2009), specific for apoptotic cells and optimized for the larval/pupal CNS (Kato et al., 2011). The entire VNC was counted, using the edges of the optic lobes as anterior boundaries. Eve^+^ cells in the larval CNS were counted automatically with DeadEasy Larval Glia software, which counts nuclear stains (see previous section; Forero et al., 2012). For Eve^+^ cell counting, the thoracic (T1–T3) and posterior tip cells were excluded because cells there are too packed together, and only the cells from abdominal segments A1–A6 were counted.

Quantification of pixel intensity was performed with ImageJ, setting fixed regions of interest over the area posterior to the morphogenetic furrow or over the morphogenetic furrow, and the mean intensity in this area was normalized over the mean intensity of a fixed region of interest over the eye disk anterior to the morphogenetic furrow.

#### Statistical analysis

Statistical analyses were performed using SPSS (IBM) and Prism (GraphPad Software). Continuous data (e.g., the number of Dcp1^+^, pJNK^+^, and Eve^+^ cells) were analyzed first for normality, using curve shape or kurtosis and skewness, and then testing the homogeneity of variance with a Levene’s test. If the Levene’s test was not significant, a one-way analysis of variance was used, and Welch analysis of variance was used if samples did not pass the Levene’s test. Multiple genotypes were compared with a single control with Dunnett’s post-hoc test or were compared with each other using Bonferroni’s multiple comparison corrections tests. For TLR signaling, data were analyzed using a two-way analysis of variance, and Dunnett’s post-hoc tests were used for multiple comparisons to NT = 0 controls. For genetic experiments, reproducibility was confirmed by the overall large population sizes and consistent results in multiple repetitions of the experiments; for cell culture data, qRT-PCR, and coimmunoprecipitations, the experiments were performed at least three times. All p-values, tests, and sample sizes are provided in figure legends, and further details are in Table S2.

### Online supplemental material

Fig. S1 shows how structural analysis of the prodomains of Spz, DNT1, and DNT2 revealed unique features in each ligand. Fig. S2 shows S2 cells expressing Toll-1, -2, -5, -7, and -8. Fig. S3 shows how DNT1 and 2 bind Toll-6 and -7 promiscuously. Fig. S4 shows the penetrance of Eve^+^ neuron number phenotypes in the embryonic CNS. Fig. S5 shows how mammalian NTs elicit signaling from TLR4 and alter the response of several TLRs to their canonical immunity ligands. Table S1 is a list of primers used, and Table S2 is a list of all genotypes, sample sizes, and statistical analysis details.

## Supplementary Material

Supplemental Materials (PDF)

Table S2 (Excel file)
