# Supplementary material for: Three-tier regulation of cell number plasticity by neurotrophins and Tolls in Drosophila
Source: J Cell Biol. 2017 May 1;216(5):1421–38. doi: 10.1083/jcb.201607098 (PMC5412559; doi:10.1083/jcb.201607098)
Supplement: Supplemental Materials (PDF) [file JCB_201607098_sm.pdf]

Foldi et al., https://doi.org/10.1083/jcb.201607098

THE JOURNAL OF CELL BIOLOGY

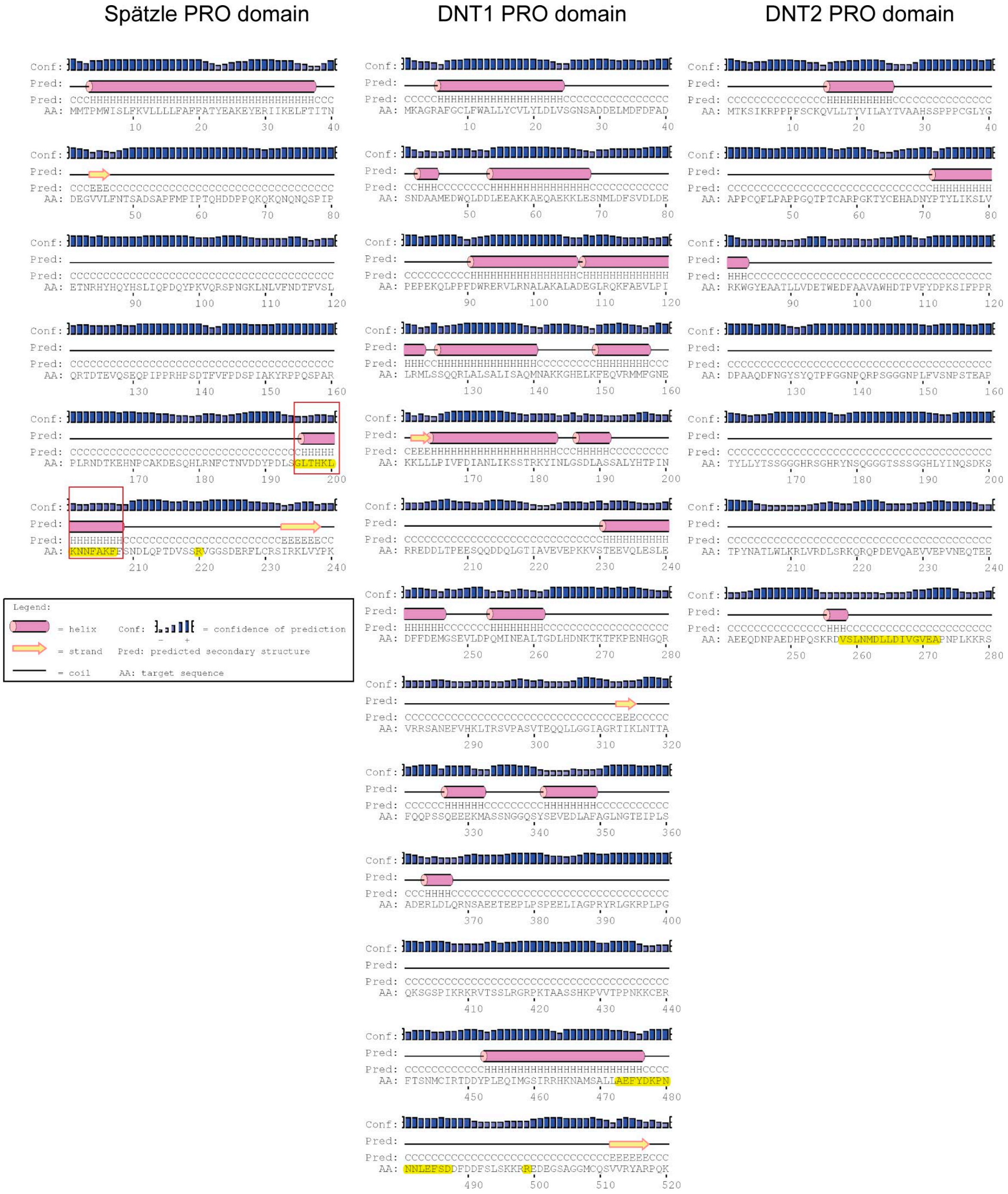

Figure S1. **Structural analysis of the prodomains of Spz, DNT1, and DNT2 reveals unique features in each ligand.** Pink barrels indicate α-helices, orange arrows indicate β strands, lines indicate coils, and sequences in yellow highlights in DNT1 and 2 indicate the putative sequences that might correspond to the Spz α-helix involved in the activation of Toll-1 (yellow and boxed). This helix is absent in DNT1 and 2, suggesting that their mechanism for receptor activation differs from that of Spz and Toll-1.

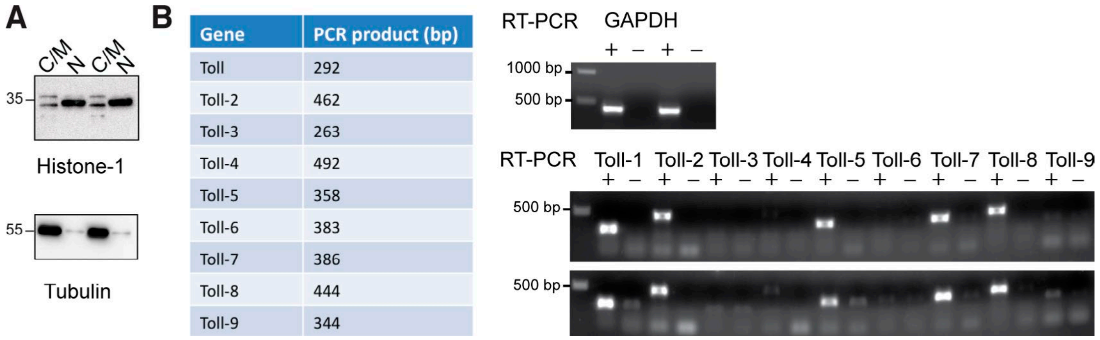

Gene	PCR product (bp)
Toll	292
Toll-2	462
Toll-3	263
Toll-4	492
Toll-5	358
Toll-6	383
Toll-7	386
Toll-8	444
Toll-9	344

Figure S2. **S2 cells express Toll-1, -2, -5, -7, and -8.** (A) Western blots showing the purity of subcellular fractionation samples for Fig. 3 C; antitubulin is restricted to the cytoplasmic/membrane (C/M) fraction, and anti–histone-1 is enriched in the nuclear fraction (N). Molecular masses are given in kilodaltons. (B) RT-PCR from S2 cells using primers to each of the Tolls showing the presence of bands corresponding with expected product sizes, which are indicated on the left. The experiment was repeated in two consecutive passages of S2 cells, shown here as top and bottom gels. GAPDH was used as a house-keeping control. Negative control reactions were performed in the absence of reverse transcription. S2 cells express Toll-1, -2, -5, -7, and -8, but not Toll-6.

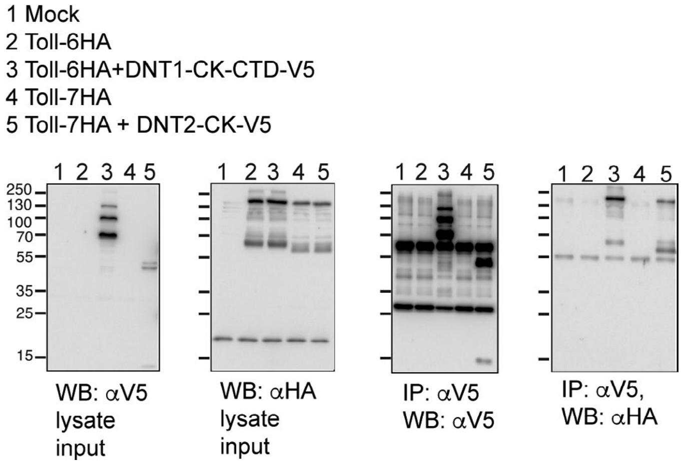

Figure S3. **DNT1 and 2 bind Toll-6 and -7 promiscuously.** Coimmunoprecipitations from S2 cells cotransfected with Toll-6–HA or Toll-7–HA and DNT1-CK-CTD-V5 or DNT2-CK-V5, respectively. The first three panels are controls. On the right, precipitating the ligands with anti-V5 brought down the receptors detected in Western blots with anti-HA. IP, immunoprecipitation; mock, no transfection; WB, Western blot. Molecular masses are given in kilodaltons.

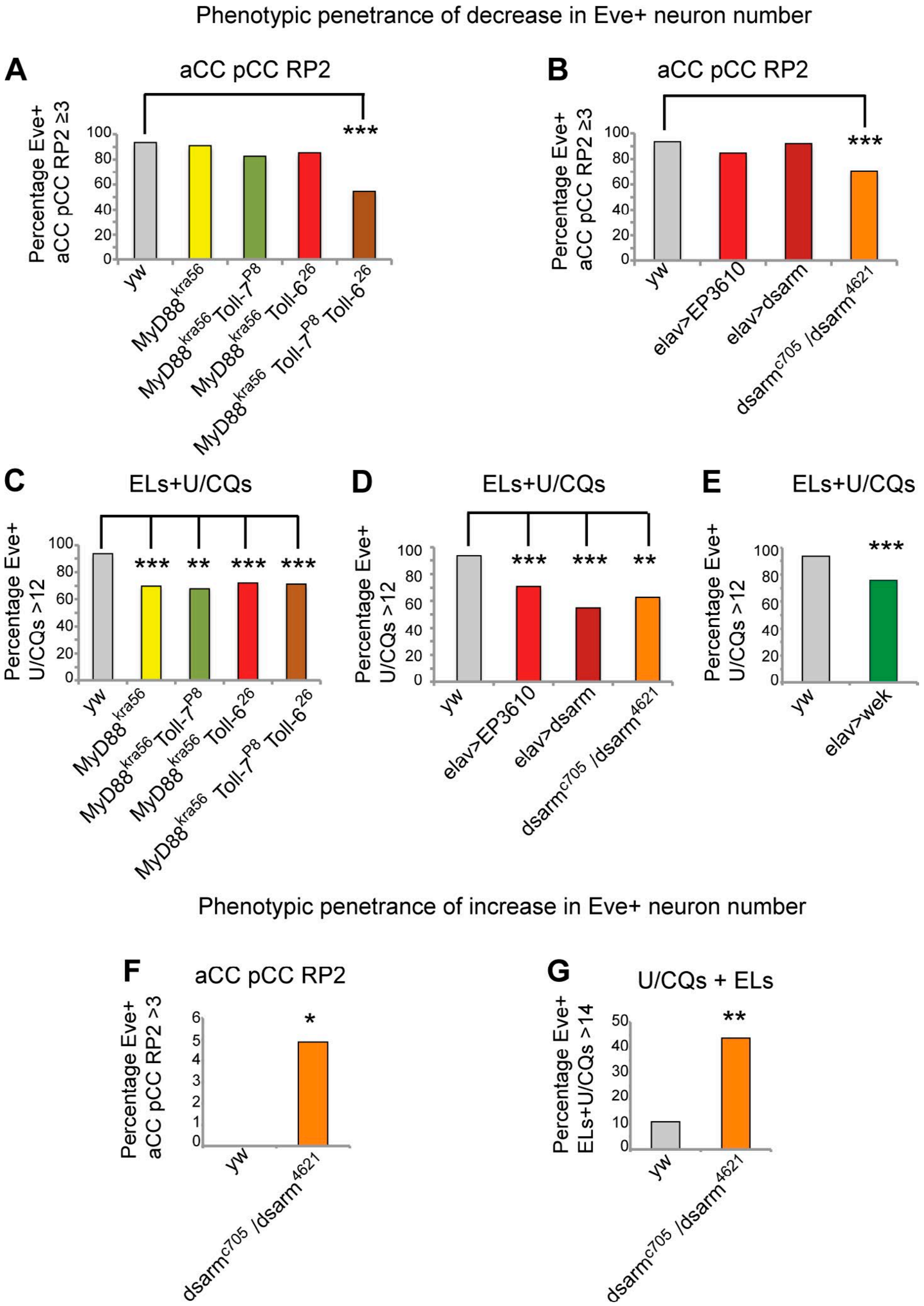

Figure S4. **Penetrance of Eve⁺ neuron number phenotypes in the embryonic CNS.** (A–E) Percentage bar charts showing phenotypic penetrance of decreases in Eve⁺ neuron numbers. (A) Dorsal anterior corner cell (aCC), pCC, and RP2 Eve⁺ neurons are lost most prominently in *MyD88 Toll-7 Toll-6* triple mutants. χ^2: P < 0.0001, Bonferroni's multiple comparisons correction; ***, P < 0.001. *n* = 52–170. (B) Loss of dorsal anterior corner cell, pCC, and RP2 Eve⁺ neurons in *dsarm* mutants. χ^2: P = 0.0002, Bonferroni's multiple comparisons correction; ***, P < 0.001. *n* = 41–170. (C) Loss of Eve lateral (EL) + U/CQ Eve⁺ neurons in MyD88, Toll-7, Toll-7 loss-of-function, double, and triple mutants. χ^2 all together: P = 0.0002, Bonferroni's multiple comparisons correction; **, P < 0.01; ***, P < 0.001. *n* = 28–119. (D) Eve⁺ Eve laterals and U/CQs are lost in embryos overexpressing *sarm* in all neurons and in $dsarm^{c705}/dsarm^{4621}$ mutants. χ^2 all together: P < 0.0001, Fisher's post-hoc exact test and Bonferroni's correction; **, P < 0.01; ***, P < 0.001. *n* = 33–106. (E) Eve laterals and U/CQs are lost in embryos overexpressing *wek* in all neurons. Fisher's exact test: ***, P < 0.001. *n* = 106 and 269. (F and G) Phenotypic penetrance of increases in the number of Eve⁺ neurons. (F) The number of dorsal Eve⁺ neurons in the anterior corner cell, pCC, and RP2 increases in *dsarm* mutant embryos. χ^2: *, P < 0.05. *n* = 41 and 170. (G) The number of Eve lateral + U/CQ Eve⁺ neurons increases in *dsarm* mutant embryos. Fisher's exact test: **, P < 0.01. *n* = 16 and 91. Asterisks on graphs refer to post-hoc multiple comparisons corrections. For statistical details, p-values, and sample sizes, see Table S2.

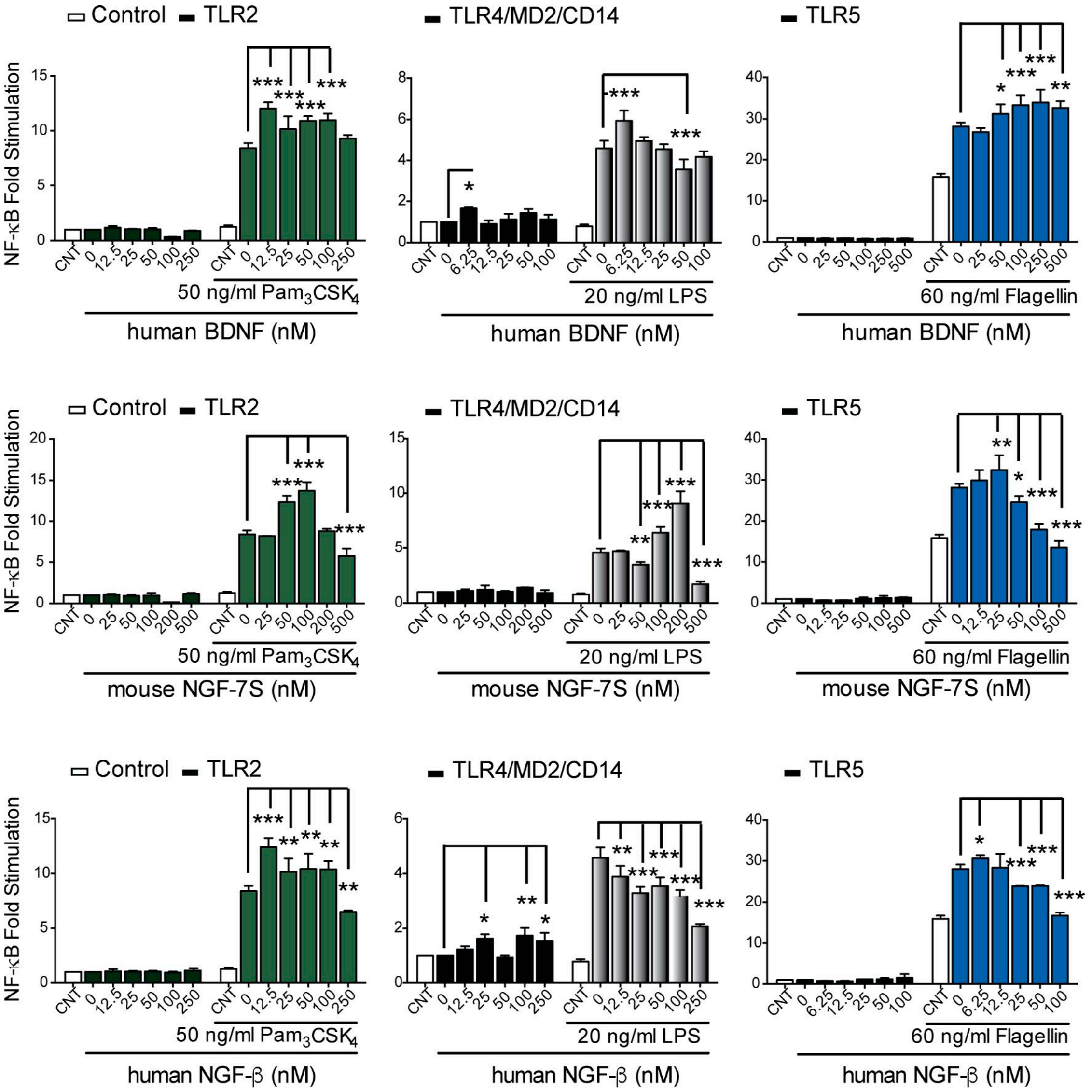

Figure S5. **Mammalian NTs elicit signaling from TLR4 and alter the response of several TLRs to their canonical immunity ligands.** HEK293T cells were transfected with TLR2, 4, and 5 and stimulated with varying concentrations of purified NGF and BDNF CK dimers, and their canonical ligands activated or modulated a luciferase reporter NF-κB signaling readout. NGF and BDNF induced signaling from TLR4 but not TLR2 or 5. Two-way analysis of variance: for TLR4 BDNF, $P < 0.0001$; for TLR4 NGF, $P < 0.0001$. Dunnett's post-hoc multiple comparison corrections to NT = 0 controls, three repeats per experiment. NGF and BDNF altered the response of TLR4, 2, and 5 to their canonical ligands involved in innate immunity. This suggests either that BDNF and NGF compete with the canonical ligands to activate TLRs, or they bind other receptors that then modify signaling by TLRs. Two-way analysis of variance: $P < 0.0001$. Dunnett's post-hoc multiple comparison corrections to NT = 0 controls, three repeats per experiment. Error bars indicate SEM. Asterisks on graphs refer to multiple comparisons corrections: *, $P < 0.05$; **, $P < 0.01$; ***, $P < 0.001$. All data are from three replicates. CNT, control; LPS, lipopolysaccharide. For statistical details and sample sizes, see Table S2.

Table S1. **List of primers used in experiments**

Figure	What for	Primer name	Sequence (5'–3')
Fig. 2 D	Site-directed mutagenesis		
		DNT2-FL R284G	CAGTCGGAAGCAGGGGCAACCCGACGA
		DNT2-FL R284G antisense	TCGTCGGGTTGCCCCTGCTTCCGACTG
		DNT2-FL R214G	TGAAGCGCTTGGTCGGGGATCTCAGTCGG
		DNT2-FL R214G antisense	CCGACTGAGATCCCCGACCAAGCGCTTCA
		DNT2-FL R221G	GTTCGAGGACAAAGGGCCAAAGTCCGGG
		DNT2-FL R221G antisense	CCCGGACTTTGGCCCTTTGTCCTCGAAC
		DNT1-FL R283G	ATGGTCAAAGAGTTCGAGGATCGGCAAACGAGTTT
		DNT1-FL R283G antisense	AAACTCGTTTGCCGATCCTCGAACTCTTTGACCAT
		DNT1-FL R499G	CCTGAGCAAAAAGAGGGGTGAAGACGAAGGCAG
		DNT1-FL R499G antisense	CTGCCTTCGTCTTCACCCCTCTTTTTGCTCAGG
Fig. 4, D and E	CRISPR mutant		
	MyD88 guide RNA	Myd88 sense BbsI	GTCGCCGAGGGAGTTATGGACTCC
		MyD88 antisense BbSI	AAACGGAGTCCATAACTCCCTCGG
Fig. 5 F	Cloning		
	dsarmPD cDNA into pDONR	attB-dSarmPD forward	GGGGACAAGTTTGTACAAAAAAGCAGGCTTCATGGGCAATCGTTTGAGCG
		attB-dSarmPD reverse	GGGGACCACTTTGTACAAGAAAGCTGGGTGCCAAAATATCATGCGCCCG
Fig. 7 C	qRT-PCR		
	RpL32	RpL32qPCRF	AAGCGGCGACGCACTCTGTT
		RpL32qPCRR	GCCCAGCATACAGGCCCAAG
	MyD88	MyD88qPCRF1	GGCTCGTTCCCTACACGATC
		MyD88qPCRR1	GAATGCTGGGAGTGGTCACC
	dsarm	dsarmqPCRF2	CGATCAAACGGCTCCCAACT
		dsarmqPCRR2	GCAGGGCATCGGGAGTTAAT
	wek	wek qPCRF1	AGAAGCCCTGTATATGCCCCG
		wek qPCRR1	TGCTTGTTAAGAATCGCCCGTGT
Fig. S2	RT-PCR		
	Toll-1	Toll-forward	CAACTGCCTACCAATCTCAC
		Toll-reverse	CTATGAACACGCCCTTTTCC
	Toll-2 (18wler)	18-Wler-forward	GCAATATCGTCACAGCCTC
		18-Wler-reverse	CACACAAACTCGTAGTCCTTC
	Toll-3	Toll3-forward	AATCACCTTCCAGCGAAAC
		Toll3-reverse	CCCAAAAACTTCAAAAACCCC
	Toll-4	Toll4-forward	CCTCATCTACTACACCTCCCTC
		Toll4-reverse	TACGCCCTCAACTCGCTATC
	Toll-5	Toll5-forward	CTTAGCGACTTACTCAAGACC
		Toll5-reverse	TCCCGAATGACACTATACCC
	Toll-6	Toll6-forward	CCTGAACGACAACCTGATAAC
		Toll6-reverse	ACTCACAGCAATGGCAAAC
	Toll-7	Toll7-forward	CTCGCACAATCGCATCACAG
		Toll7-reverse	GACGCAGACCACTCAAAGGA
	Toll-8	Toll8-forward	GCAGATCCTTAACCTGTCCC
		Toll8-reverse	TTCCTCACCAAATCCACCC
	Toll-9	Toll9-forward	CCCCTACCTATCCTACAACATC
		Toll9-reverse	AATCCAATCGCTCAAAGTCC

Table S2 is a separate Excel file and contains a list of all genotypes, sample sizes, and statistical analysis details.
